# Radiomic Characterization of Breast Tissue from Breast CT Images Obtained with Synchrotron Beams

**DOI:** 10.3390/tomography12090124

**Published:** 2026-08-29

**Authors:** Marlen Perez-Diaz, Ariel Fernandez-Pirez, Luca Brombal, Renata Longo, Anton Maksimenko, Caroline Kachana Pwamang, Stevan Vrbaski, Luigi Rigon

**Affiliations:** 1 Division of Trieste, National Institute for Nuclear Physics (INFN), 34149 Trieste, Italyrenata.longo@ts.infn.it (R.L.); luigi.rigon@ts.infn.it (L.R.); 2 TRIL Program, The Abdus Salam International Centre for Theoretical Physics (ICTP), 34151 Trieste, Italy; 3 Center for Research and Advanced Studies of the National Polytechnic Institute, Mexico City 07360, Mexico; arielfdez26@gmail.com; 4 Department of Physics, University of Trieste, 34127 Trieste, Italy; pwamangc@yahoo.com; 5 Australian Synchrotron, ANSTO, Clayton, VIC 3168, Australia; antonm@ansto.gov.au; 6 Department of Medical Physics, University of Ghana, Legon, Accra G4-489-4642, Ghana; 7 Nuclear Regulatory Authority, Accra GE-291-5715, Ghana; 8 Faculty of Medicine, University of Novi Sad, 21137 Novi Sad, Serbia; stevan.vrbaski@mf.uns.ac.rs

**Keywords:** synchrotron radiation, breast computed tomography, breast cancer, radiomics

## Abstract

**Background/Objective:** Radiomics offers a powerful, non-invasive approach for extracting quantitative features to predict lesion phenotypes. This study performs a radiomic characterization of breast tissue using synchrotron radiation breast computed tomography (SR-bCT). **Method:** Four mastectomy samples were imaged at the Australian Synchrotron (ANSTO) using five energies (25, 32, 35, 40, and 60 keV). From 10 slices per energy for each sample, 1546 regions of interest (ROIs) were extracted across four tissue subtypes: fatty, glandular, fibrous, and microcalcified. Using Pyradiomics, 93 features were initially calculated and then reduced to 34, eliminating highly correlated variables to reduce redundancies. Seven linear regression models and a discriminant analysis evaluated subtype tissue separation and radiomic characterization across individuals and combined samples and energies with a good fit (0.81 ≤ R ≤ 0.98). **Results:** The study identified six possible robust exploratory candidate features independent of sample variability and energy levels, whose mean values are significantly different among the four tissue subtypes (clusters, *p* < 0.001). The selected candidate features were 90th Percentile, Kurtosis, Skewness, GLCM IDM, GLDM LDE, and NGTDM Coarseness. These metrics successfully differentiated all tissue pairs (microcalcifications/fat, microcalcifications/gland, microcalcifications/fiber, fat/gland, fat/fiber and gland/fiber), with *p* < 0.05 inside the most general regression model and *p* < 0.0001 with the linear discriminant separation in clusters. **Conclusions**: Findings indicate that some first-order and second-order texture metrics reflecting global dependencies remain stable across experimental conditions. Conversely, fine-texture metrics are highly sensitive to sample energy changes, limiting their generalizability. These results align with successful biomarkers in mammography and validate the potential of radiomics in SR-bCT characterization.

## Simple Summary

Radiomics has been successfully employed in very diverse types of medical images. Various radiomic metrics have played a very important role in the characterization of tumors, malignancy degree, extent, and disease prognosis. To date, no radiomic study using synchrotron beam breast tomography images (SR-bCT) have been published. Therefore, this is a preliminary work, in which six radiomic metrics were identified as possible exploratory candidate features to separate tissue pairs (microcalcifications/fat, microcalcifications/gland, microcalcifications/fiber, fat/gland, fat/fiber, and gland/fiber) independently of the sample and acquisition energy. Based on the metrics selected and on what is known about them in the scientific literature in other types of imaging, this work opens new research hypotheses for radiology and oncology to study the degree of malignancy of breast tumors, evaluate the response to chemotherapy treatments, or differentiate breast lesions between solid tumor, cyst or necrosis, using SR-bCT and radiomics.

## 1. Introduction

In the last decade, radiomics has experienced exponential growth in breast oncology, having been applied for detection, diagnosis, and prediction of treatment response. It has been successfully employed using images from mammography, tomosynthesis, computed tomography (CT), magnetic resonance imaging (MRI), ultrasound, elastography, and molecular imaging from nuclear medicine, including positron emission tomography (PET) and PET-CT [1,2].

From all the aforementioned imaging techniques, it has become evident that various radiomic metrics play a very important role in the characterization of tumors, their degree of malignancy, extent, and prognosis of the disease [3]. First-order metrics such as the mean, median, and percentiles are used to measure contrast enhancement or cell density and therefore help to differentiate between solid, cystic, or necrotic tissues. Standard Deviation, Variance, and Interquartile Range have proven to be good indicators of aggressive tumors as they correlate well with the fact that these tumors tend to be more heterogeneous due to the presence of internal bleeding, necrosis, or different cell densities. High Variance has been correlated with a worse prognosis. Other metrics, such as Skewness and Kurtosis, have been identified as biomarkers for predicting treatment response, as these metrics change markedly when a tumor becomes more homogeneous after chemotherapy [3].

However, while first-order metrics can be robust and easy to calculate, they are not sufficient on their own to discriminate among many patterns [4]. In fact, two images can have the same histogram, i.e., the same distribution of gray levels, and yet be visually very different. Therefore, second-order metrics, which characterize texture and its spatial relationships, are also used.

Malignant tumors have been found to typically have a heterogeneous structure, while benign tumors are more homogeneous. Their textural difference has been identified using metrics derived from the Gray Level Co-occurrence Matrix (GLCM), which has helped identify tumors at early stages. Among these, high Energy and low Homogeneity, for example, have been used to indicate malignancy. The same has been demonstrated for high Contrast and Entropy values and a low Inverse Difference Moment (IDM) [5].

Gray Level Run Length Matrices (GLRLMs) have also helped demonstrate that tumors with high heterogeneity are associated with aggressive growth, poor prognosis, and worse therapeutic outcomes [4,5]. They have been correlated with prognostic factors such as the Ki67 proliferation index, receptor status (ER, PgR, HER2+), and histological grade. Gray Level Dependency Matrices (GLDMs), for their part, have been investigated to differentiate between the degree of malignancy and the type of breast tumor [4,5].

Within Neighboring Gray Tone Difference Matrices (NGTDMs), high Busyness reflects greater biological heterogeneity [5] and indicates areas with greater tumor aggressiveness. Furthermore, NGTDM-based radiomic textures can distinguish between benign and malignant lesions, often showing highly complex patterns in invasive tumors. Therefore, they are biomarkers that allow monitoring changes in tumor structure over time or after therapy, overcoming the limitations of tissue sampling (traditional biopsy) [4]. Furthermore, their use in differentiating molecular subtypes (such as triple-negative or HER2+) is being investigated. Low Coarseness values have also been associated with more aggressive or malignant tumors, while high Contrast levels suggest a high difference between pixels, often correlated with high-grade tumors.

It should be noted that the usual application of radiomics has been in the study of tumors. Although density thresholds (Hounsfield in CT or gray levels in X-rays) are generally able to identify different tissue subtypes with relative ease, it makes sense to use radiomics in specific scenarios where the anatomy is complex, for example, to characterize microcalcifications. That is, not to see if a calcification is present (which may be obvious if the image quality is good), but rather to analyze its subtle texture and morphology. Radiomics has proven to be a good method for differentiating whether a group of microcalcifications is benign (typically more homogeneous) or malignant (pleomorphic), something that may escape the trained eye of the radiologist [6].

Also, in stromal analysis, radiomics allows for the quantification of parenchymal complexity. Beyond simply determining whether a breast is dense, radiomics can measure the spatial organization of the fibers and glands. Furthermore, with respect to cancer risk, it is used to identify risk signatures in healthy tissue [7]. For example, a gland with high entropy may indicate a microenvironment more prone to tumor development. Another application is in differentiating infiltrating tumor, which may have intensities similar to the gland [8]: second-order radiomics reveal that, although the brightness on the image is the same, the grain pattern of normal fibers is distinct from the disorganized and dense pattern of infiltrating tumor tissue.

Although studies have shown that synchrotron beam-based tomographic images have a much finer granularity (spatial resolution) and a higher signal-to-noise ratio (SNR) than their conventional CT counterparts [9], to our knowledge, no study has yet been identified that properly characterizes the radiomics of breast tissue using tomographic images obtained from synchrotron radiation.

Synchrotron radiation breast computed tomography (SR-bCT) is an advanced technique for breast imaging. Unlike conventional bCT, which uses divergent and polychromatic X-rays, synchrotron imaging takes advantage of parallel, monochromatic, high-intensity, and spatially coherent X-ray beams. Therefore, SR-bCT shows edge enhancement due to phase contrast: when the beam crosses a boundary (for example, the boundary between a tumor and fat), the tissue edges are much sharper than in a standard CT scan [10,11,12,13].

Thus, phase contrast can allow visualization of tumors that have a density extremely similar to surrounding tissue, something impossible with standard CT. Moreover, it reveals elements such as the borders of an infiltrating tumor or the fine architecture of ducts, with contrast-to-noise ratios up to 10 times greater than standard bCT [14].

Also, by matching an SR monochromatic beam with suitable detectors, spatial resolution on the order of tens of microns can be achieved, with an average glandular dose equal to or less than that of conventional 2D mammography [12].

In addition to the above, the use of synchrotron radiation imaging would be an ideal scenario for linking it with radiomics for two other reasons: First, the absence of beam hardening (monochromatic beams) artifacts and geometric distortion (parallel beams), in contrast to common X-ray tubes [9]. This makes radiomic texture metrics more reproducible. Second, texture features can be extracted on a very small scale (microradiomics: 50–100-micron voxels), which can help differentiate between normal dense fibrous tissue and carcinoma in situ.

It should be added that, due to the high spatial resolution, these studies could serve as a reference for validating radiomics algorithms in hospital bCT. The high quality of synchrotron data can be used as a “ground truth” that allows for training radiomics models in clinical bCT, ensuring that the metrics extracted in the hospital truly correspond to the tissue architecture observed at the synchrotron. For this reason, the objective of this work is to conduct a preliminary study to find robust radiomics biomarkers capable of discriminating the radiomic boundary between fat, low-density gland (stroma), denser fiber, and microcalcifications, using SR-bCT images, which could serve as a reference for studies of conventional bCT scans and for future normal/abnormal, malignant/benign classification models in various breast pathologies.

Finally, another justification for robust radiomic characterization is the negative influence of metric redundancy on detection or classification models. Any radiomic metric calculation platform offers hundreds or even thousands of computational variables. Considering that radiomic characterization is a first step in developing classification tasks that often involve artificial intelligence, reducing the size of the radiomic vector is a critical step in CT analysis to ensure that models can generalize their predictions to new patients. It has been observed for various imaging techniques that many radiomic features are highly correlated with each other [15]. For example, several texture descriptors may be measuring the same physical phenomenon in the image. Redundancy does not provide new information and can confuse the algorithm, giving undue weight to a single type of information. A model that relies on 5 or 10 robust biomarkers is much easier to validate and more useful to radiologists than one that uses hundreds of abstract variables. Selecting the most stable and relevant features allows us to identify which aspects of breast morphology or texture are well represented by generalizable metrics [15].

## 2. Materials and Methods

### 2.1. Samples

Four freshly excised, unfixed total mastectomy breast specimens were used. The study was approved by the Human Research Ethics Committee (project number: CF15/3138-2015001340) after obtaining written informed consent from the patients.

Specimens were obtained after relevant material had been removed for anatomopathological analysis. Each sample was placed inside a sealed plastic cylinder measuring 110 mm in diameter. They were mounted vertically for scanning, with the anatomical axis aligned from the nipple to the pectoralis major muscle [16]. The specimens were characterized as follows:

Sample 1: A 66-year-old patient who underwent a left mastectomy and received chemotherapy for invasive ductal carcinoma, which was positive for human epidermal growth factor receptor 2 (HER2+). Histopathological analysis revealed a treated tumor bed with a minimal focus of residual invasive carcinoma (0.6 mm) and associated high-grade ductal carcinoma in situ (DCIS).

Sample 2: A 44-year-old patient with microinvasive carcinoma associated with high-grade DCIS who had undergone chemotherapy. At the time of mastectomy, a residual focal area of high-grade malignancy of 0.4 mm remained, along with widespread stromal and ductal calcifications.

Sample 3: A 77-year-old patient who underwent a right mastectomy for high-grade DCIS. The histopathological study revealed an extensive (nearly 160 mm) area of high-grade DCIS with comedonal necrosis and two microcalcifications. Within this background, small foci of microinvasive carcinoma, measuring 0.1–0.2 mm, were also detected. The tumor was ER3+ and PR2+.

Sample 4: A 53-year-old patient with a history of left breast cancer, for which she underwent wide excision, sentinel lymph node biopsy, and radiotherapy. She was diagnosed with 15 mm high-grade DCIS.

### 2.2. Radiation Exposure and Image Acquisition

**Figure 1:**
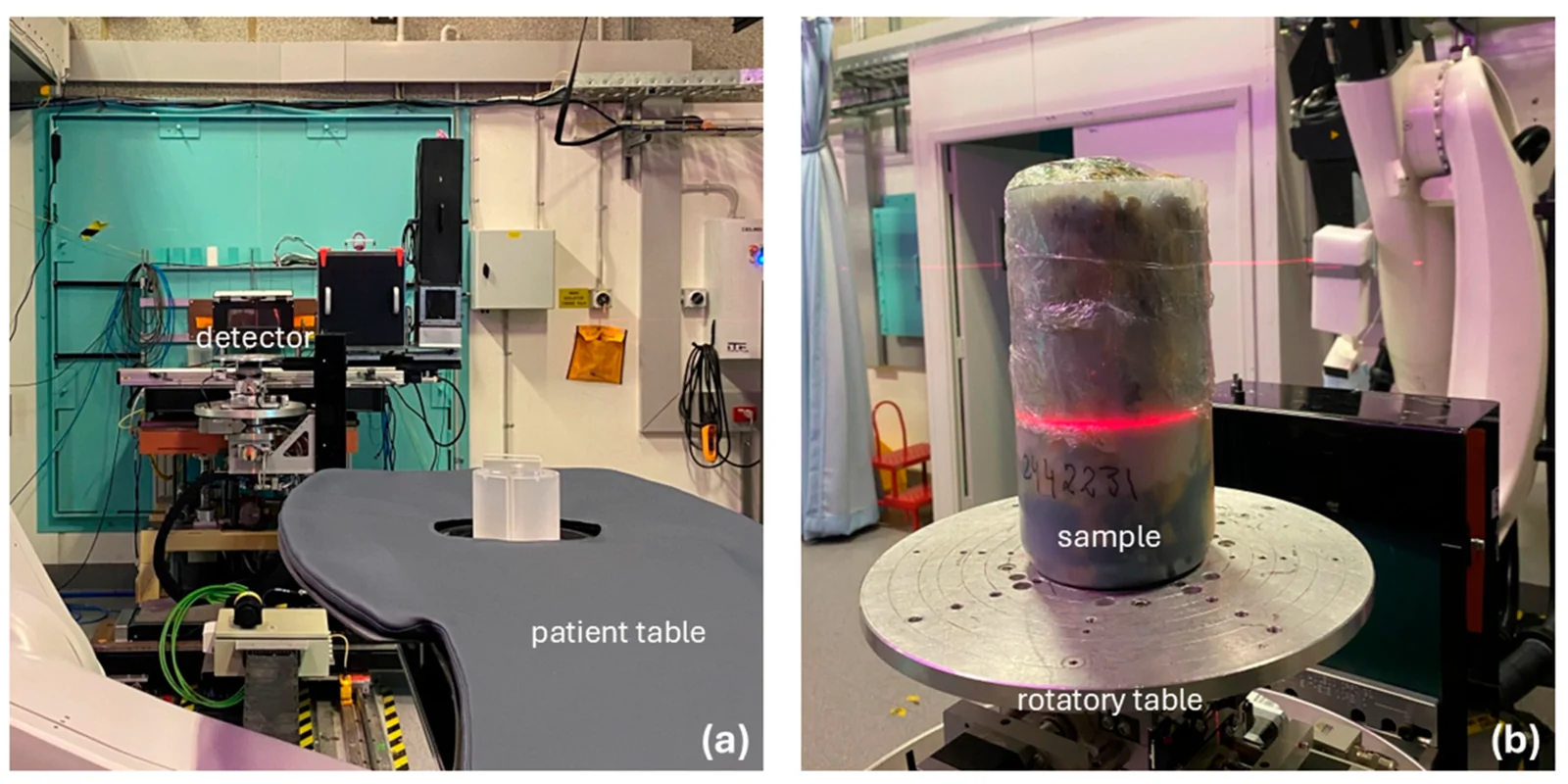
(**a**) Acquisition facility at ANSTO, in the standard configuration for patient scanning; the patient table is visible in the front, and the CdTe photon counting detector is in the back. (**b**) The acquisition setup used for this experiment: one of the samples is positioned on the rotatory table and a laser reference beam is used for alignment.

Image acquisition was performed at the ANSTO Australian Synchrotron facility, at the Imaging and Medicine Beamline (IMBL) [17], which provides a monochromatic X-ray beam at 140 m from the source. A CdTe photon counting detector with a pixel size of 75 μm was used (Figure 1a). For tomographic acquisition, each sample in the plastic cylinder was placed on a platform that rotated at a constant speed of 4.7° s^−1^ while 4800 projections were acquired in a 180° angular range. In this case, the typical patient support in the facility was replaced by a rotating table for placing and scanning the samples (Figure 1b).

The scan time was 38.4 s. Between 8 and 12 scans were performed at different vertical positions of the table to capture the full volume of each sample, which corresponded to a height between 90 and 150 mm. The total scan time was between 5 and 8 min. To employ the propagation-based phase-contrast imaging technique [18], the samples were placed 6 m from the detector.

Breast CT images for each sample were acquired at different energies in a range from 23 to 60 keV [19]. For comparative purposes in this study, only the images acquired at energies of 25, 32, 35, 40, and 60 keV, which were common to all four samples, were used. The Mean Glandular Dose (MGD) was estimated in the range of 23–29 mGy at the minimum energy of 25 keV, down to 9.4–11 mGy at the maximum energy of 60 keV. Figure 1 shows a picture of the general setup and one of the samples on the acquisition table.

### 2.3. Raw Data Processing and Image Reconstruction

Processing was performed following a standard pipeline on the IMBL online computing platform to obtain the final three-dimensional dataset of the whole scanned samples [20]. Projections of each vertical scan were normalized via a flat field procedure, stitched together to form full-sample height projections, and further processed to fill the gaps between the detector modules. A conventional Homogeneous Transport of Intensity (TIE-Hom) phase-retrieval filter was then applied to each projection [21], where the delta/beta ratio refers to a glandular/adipose tissue interface and is extracted from tabulated data [22]. After phase-retrieval, the projections of different scans are vertically stitched and tomographic reconstruction is performed via a GPU-accelerated filtered backprojection (FBP) algorithm with a Shepp–Logan filter [13,23].

**Figure 2:**
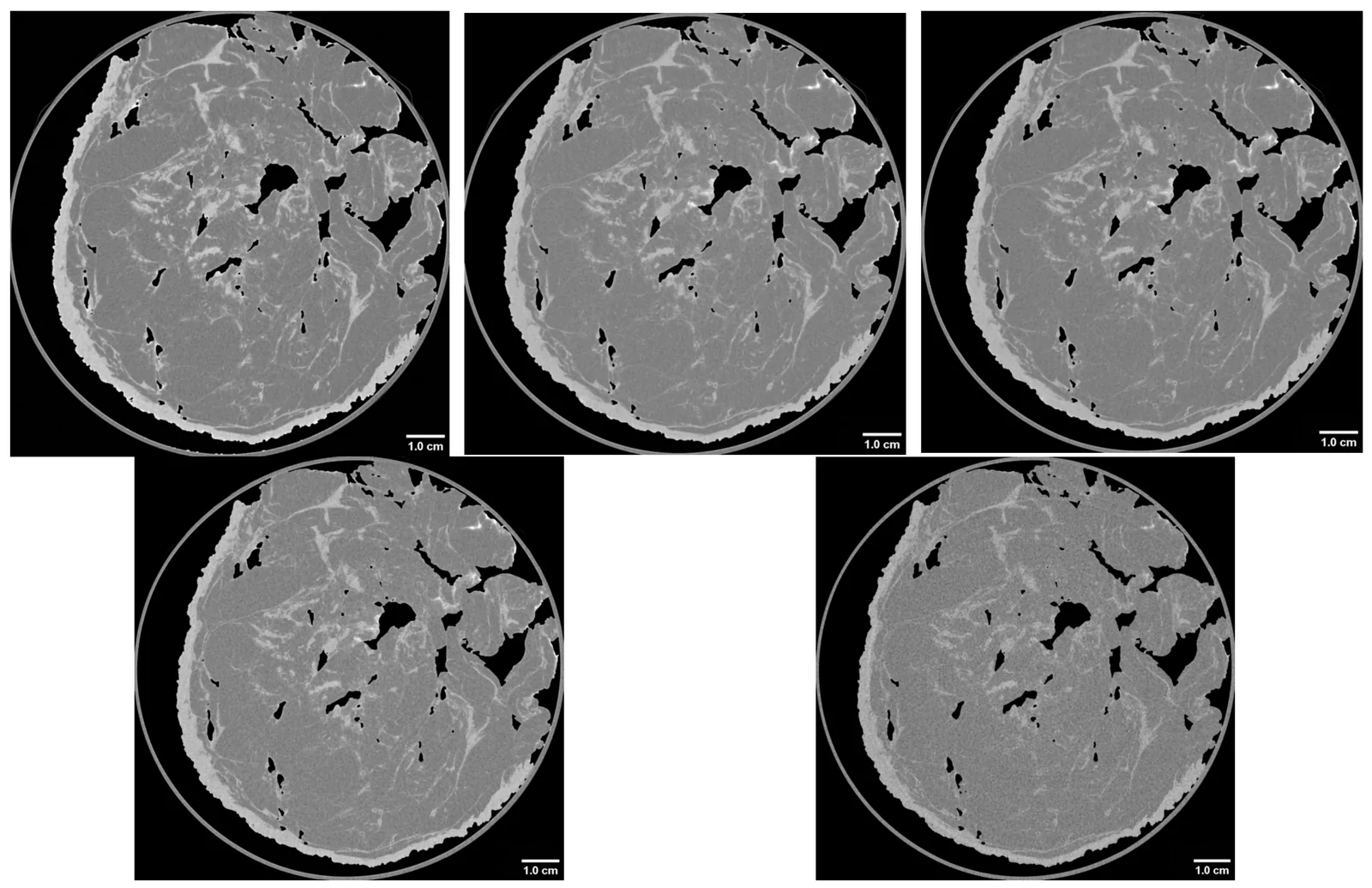
Slices of sample 3 at 25, 32, 35, 40 and 60 keV, respectively. As energy increases, detail visibility is diminished due to the lower contrast and increased noise.

Figure 2 shows an image (2376 × 2376 pixels) of the same slice of a sample at the five energies chosen for this work.

### 2.4. Radiological Report

**Figure 3:**
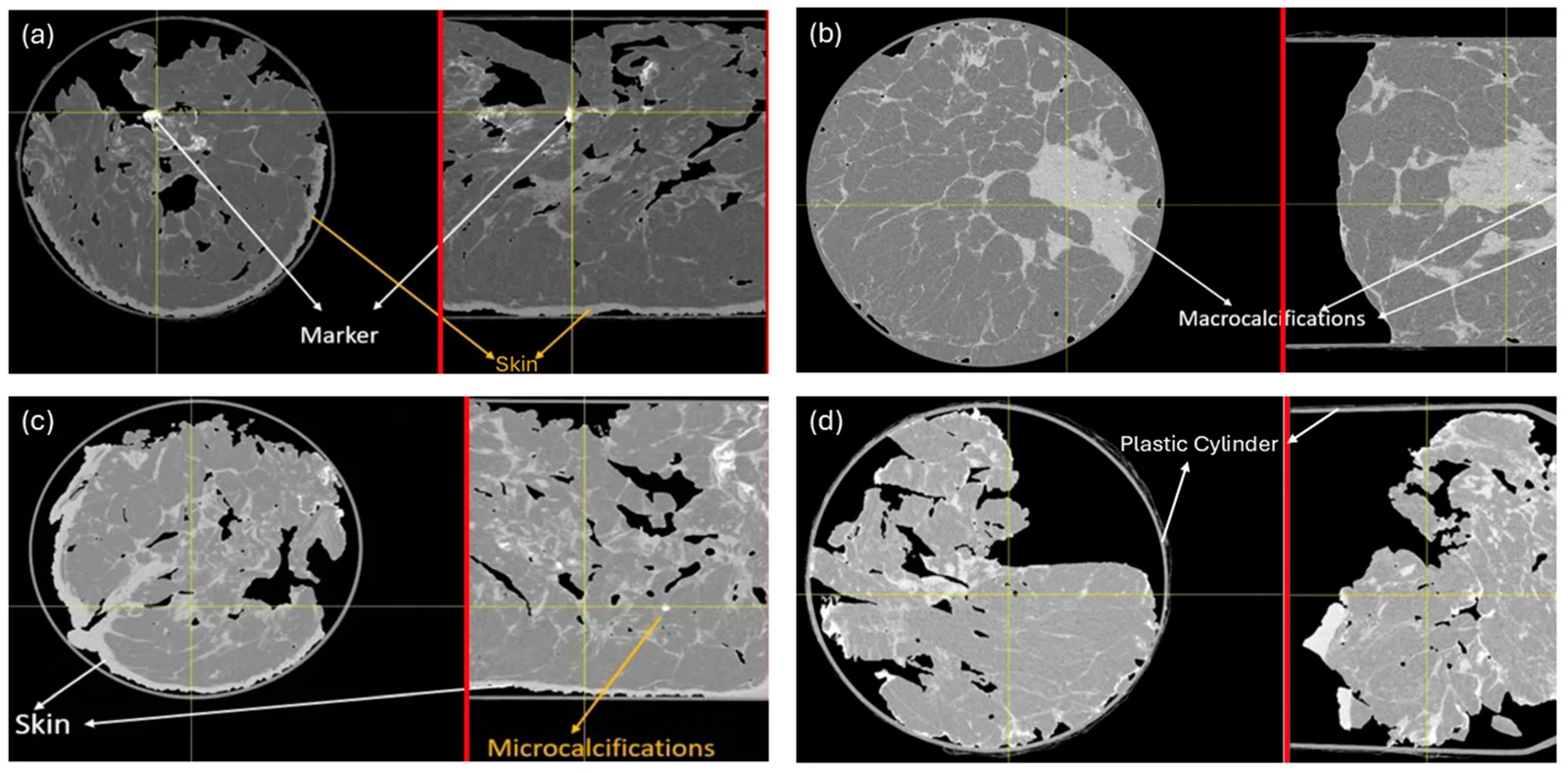
(**a**–**d**) Representative views of samples 1–4, respectively. For each sample, an axial slice and an orthogonal section are shown. Arrows indicate specific details. Thin yellow lines indicate the positions of the horizontal and vertical planes that correspond to axial slices and orthogonal views.

Figure 3a–d shows typical axial and coronal sections of the four samples. In all cases, there was consistency between the radiological report, based on the SR-bCT scans of each sample and prepared by an expert medical specialist, and the histological report. No tumor mass was observed in the images or in the histology of the samples, which all exhibited ductal carcinoma. Two specimens also had microcalcifications, concomitant with DCIS. Microcalcifications were present in 124 glandular regions of sample 2 (some of them visible in Figure 3b), while sample 3 had only 2 microcalcifications (one visible in Figure 3c). A surgical marker in the area where the tumor was located was still present in the images of sample 1 (Figure 3a). Regarding sample 4, the tissue appears highly fragmented due to surgical resection (Figure 3d).

### 2.5. Radiomics Experiments

**Figure 4:**
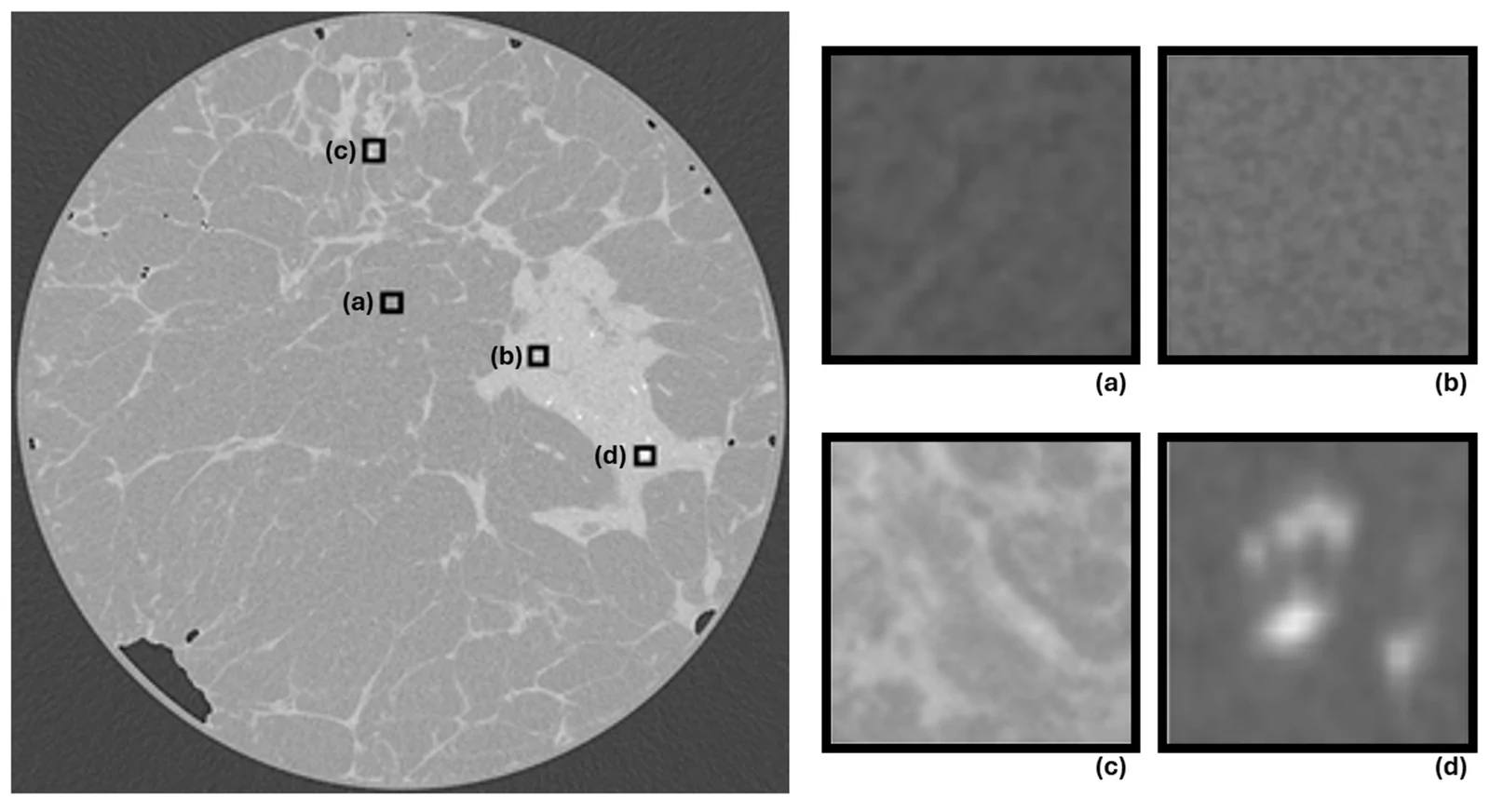
ROIs in (**a**) fat, (**b**) gland, (**c**) fiber and (**d**) microcalcifications.

Ten tomographic slices were randomly selected from each sample. Each slice was scanned at five energies (25, 32, 35, 40, and 60 keV), and for each slice, ten 100-pixel square (10 × 10) regions of interest (ROIs) were selected, located within tissue subtypes characterized by fat, the gland (considering only low-density areas associated with the stroma), fiber (denser tissue overlying the gland, including bands, lines, lobules, and ducts), and microcalcifications (over fat or stroma), for a total of 1546 ROIs. Although two or more tissue subtypes could overlap within the same ROI, as previously described, the small size of each ROI ensured that one subtype always predominated (at least 60% in microcalcifications, 70% in fiber, 80% in stroma, and 90% in fat). To avoid bias in ROI placement, the ROIs were automatically reproduced for the same slice at different energies. All selected ROIs were verified by a second investigator, with complete agreement between the two reviewers, and all doubtful ROIs were reviewed with an expert radiologist. Figure 4 presents an example of an ROI in each tissue subtype and an SR-bCT slice with four ROIs, one in each tissue subtype.

In the 1546 ROIs, 93 first- and second-order radiomic metrics were calculated using the Pyradiomic platform v3.1.0 [24] with Python 3.9.13, NumPy 1.26.4, SimpleITK 2.3.1, and PyWavelets 1.6.0. Each reconstructed synchrotron CT slice was processed independently as a two-dimensional image. TIFF image arrays were converted to 32-bit floating-point SimpleITK images, and binary masks were generated with pixels inside the ROI assigned a value of 1 and background pixels assigned a value of 0. No image-intensity normalization, outlier removal, or intensity re-segmentation was applied. Gray-level discretization was performed using a fixed bin width of 25. Images were analyzed at their native sampling grid without resampling; consequently, no interpolation was applied during feature extraction, although B-spline interpolation was retained as the default configured interpolator. A neighborhood distance of one pixel was used for the applicable texture matrices. The analyzed radiomic feature classes comprised first-order statistics, GLCM, GLRLM, GLSZM, GLDM, and NGTDM. The 93 features considered in the statistical analysis were those calculated from the original image representation.

Next, Pearson correlation coefficients were calculated for the original 93 radiomic features. A threshold of |r| ≥ 0.60 was used to identify strongly correlated features. Within each radiomic family, when highly correlated metrics essentially represented the same radiomic aspect according to their PyRadiomics definitions, one representative feature was retained, and the redundant feature(s) were removed. For example, mean absolute deviation and robust mean absolute deviation showed a Pearson correlation of approximately 0.90 and describe closely related aspects of the data; therefore, mean absolute deviation was removed and robust mean absolute deviation was retained. The same principle was applied to the texture families. For example, GLCM Contrast and GLCM IDM were retained because they represent different textural properties, whereas IDM and IDMN, which are highly correlated and represent closely related concepts, were reduced to a single representative feature (IDM). Similarly, between GLRLM GLNU and GLNU Normalized, GLNU was retained, while between GLDM LDE and HGLE, LDE was retained because of its high correlation with HGLE and similar interpretation.

In this part of the work, the reduction from 93 to 34 features was not intended to eliminate every statistical correlation present in the dataset. Rather, it was designed to remove clear intra-family redundancy among metrics that essentially describe the same radiomic characteristic, while preserving complementary radiomic information.

The total group of 93 original metrics was then reduced to 34, resulting in 90th Percentile, Interquartile Range, Median, Robust Mean Deviation, Root Mean Square, Skewness, Kurtosis, Entropy, Total Energy, GLCM Cluster Shade, GLCM Correlation, GLCM Cluster Prominence, GLCM Contrast, GLCM Maximum Probability, GLCM Difference Entropy, GLCM Difference Variance, GLCM Difference Moment (IDM), Informational Measure of Correlation (1-IMC1), Join Entropy, Sum Entropy, Dependence (non-uniformity), Small Dependence Emphasis, Grey Level Non-Uniformity, Large Dependence Emphasis (LDE), Grey Level Non-Uniformity, Variance, Long Run Emphasis, Run Entropy, Run Length Non-Uniformity, Short Run Emphasis, Busyness, Coarseness, Complexity, and Contrast.

Mean ± Standard Deviation values were obtained for each metric in each tissue subtype (group) by sample and overall. Next, using JAMOVI 2.7.16 [25], seven linear regression models were constructed to separate tissue subtypes: one model with sample 1 at low energy (73 ROIs) (25 keV) and another at high energy (60 keV), and the same for sample 2 (94 ROIs); one model for sample 1 at all energies (413 ROIs) (25, 32, 35, 40, and 60 keV) and the same for sample 2 (378 ROIs); and finally, one model for all four samples at all energies (1546 ROIs). This refined the radiomic metrics that were independent of the sample and energy in recognizing tissue subtypes, and which are therefore candidates to be biomarkers for this type of study.

However, while multiple linear regression models are implemented as an exploratory step to identify potential radiomic features associated with tissue subtype, considering tissue subtype as a numerical ordinal variable (1 = fat, 2 = gland, 3 = fiber, 4 = microcalcification), it is recognized that this approach assumes equal spacing between consecutive subtypes and treats differences as homogeneous, a biologically improbable assumption (e.g., the radiomic distance between fat and gland is not necessarily equal to that between fiber and microcalcification). Nevertheless, this simplification allows for the initial classification of features based on their ability to separate tissue subtypes along a density/texture gradient.

Due to the above concern, we have performed an additional unsupervised clustering analysis as a sensitivity analysis to investigate whether the feature-selection results could be strongly influenced by the original supervised structure of the data. The results are provided in the Supplementary Information. So, given the limitations of the approach for feature selection, significant features identified via linear regression are subsequently validated using linear discriminant analysis (LDA), which does not assume ordinality or equal spacing and is the appropriate method for multiclass classification with categorical dependent variables.

Classification performance was assessed using accuracy, balanced accuracy, macro-averaged precision, recall (sensitivity), specificity, and F1-score, calculated from the confusion matrix according to standard definitions [26]. Each metric is calculated based on the classic definitions of true positives, false positives, true negatives, and false negatives. For each tissue subtype, TP (true positives) represents the number of correctly classified ROIs belonging to that subtype, TN (true negatives) represents the number of ROIs from other subtypes correctly classified as not belonging to that subtype, FP (false positives) represents ROIs from other subtypes incorrectly classified as that subtype, and FN (false negatives) represents ROIs belonging to the subtype that were incorrectly classified as another subtype.

To further assess the potential influence of specimen-level dependence, we performed a leave-one-specimen-out cross-validation analysis using specimen-level separation, in which the classification model was evaluated while keeping specimens separated between training and validation subsets. These results are included in the Supplementary Information.

## 3. Results

To illustrate the results under reduced data conditions, Table 1 includes the Mean ± Standard Deviation (SD) value of the 34 final variables included in the tissue subtype separation analysis for sample 2, considering all energies.

However, although the order of the metric values remained consistent across all other samples, not all metrics were significantly different within the constructed models, nor were they the same for all models. Differences existed based on energy levels and sample types. Specifically, many calculated radiomic metrics were found to be insensitive to changes in energy levels and sample types when constructing a model to separate the four tissue subtypes. Table 2 presents a comparative summary of significant variables (*p* < 0.05) in each model. A comparison is also included when all samples are included at all energy levels.

In sample 1 at low energy, only a few second-order metrics of texture and spatial dependencies were significant. For sample 2, although the mean values of the variables remained in the same order as in sample 1 at low energy, the significant variables yielded textural and spatial dependency information; first-order statistics were also present.

Sample 1 at high energies indicates sensitivity to the intensity distribution and to a few spatial dependencies. In sample 2 at high energies, the significance of first-order metrics, texture, and local dependencies was much more diversified, which corresponds to the greater tissue complexity of that sample.

When sample 1 was expanded to 413 ROIs of data, including all five energies, the importance of first-order metrics and some second-order texture and spatial dependency metrics was confirmed, suggesting a change in the sensitivity of the microstructure to the intensity distribution. In other words, intermediate energies provided combined first- and second-order sensitivity for texture and spatial dependence, although second-order significance was concentrated in only a few metrics.

When the sample size (energy range) increased in sample 2, the radiomic analysis converged toward a smaller subset of first-order metrics, as well as large-scale spatial dependencies and global measures of non-uniformity, while fine-texture metrics lost significance.

The previous analysis shows that there is a core set of metrics that was independent of energy in differentiating tissue subtypes across all samples. Regarding the comparison between samples, the results indicate that the more complex GLCM metrics were more dependent on the specific spatial distribution of each sample, while the first-order metrics showed greater consistency in the face of changes. As shown in Table 2, models 1 through 6, by including only partial data, allow for the detection of metric behavior in response to variations in energy and sample type, and the identification of those that are not robust to these changes.

When all samples and all energies were included to construct a general model, an intersection of metrics was obtained that was relevant under multiple diverse conditions. Table 3 shows the significant metrics (*p* < 0.05) in the general model, which involves the complete dataset (1546 ROIs). The results were corrected using the Benjamini–Hochberg (FDR) method to limit the expected proportion of false positives (Type I errors) [27]. The significance of the models associated with partial datasets is provided in the Supplementary Information S1.

The combined results from Table 2 and Table 3 are interpreted as follows: The following metrics did not determine the separation into tissue subtypes: Interquartile Range, Robust Mean Absolute Deviation, GLCM Cluster Shade, GLCM Cluster Prominence, GLCM Contrast, GLCM Dependence Non-Uniformity, and GLRLM Run Entropy. These were not significant for any of the constructed models (*p* > 0.05).

A group of metrics showed very weak discrimination and is therefore not considered robust for the task: Median, Root Mean Square, Entropy, GLCM Difference Entropy, GLCM Difference Variance, GLRLM Grey Level Variance, and GLRLM Long Run Emphasis. These metrics appear to depend on the specific microstructure at certain energies or samples and were not significant in the overall model (*p* > 0.05).

There is another group that is moderately robust: Total Energy, GLCM Correlation, GLCM Maximum Probability, GLCM IMC1, GLDM Small Dependence Emphasis, GLCM Joint Entropy and Sum Entropy, and GLDM Grey Level Non-Uniformity. These appear significant within the overall model (*p* < 0.05), but when analyzed in smaller samples, they were not always representative, as they showed fluctuations by energy level and sample.

Finally, we found the robust metrics, which appeared significant in most of the models with reduced data for most energies and in the overall model: First order: 90th Percentile, Kurtosis, Skewness. Second order: GLCM IDM, GLDM LDE, and NGTDM Coarseness.

These results reduce the radiomics range to only six significant metrics for differentiating tissue subtypes from the SR-bCT images used in this experiment. Table 4 shows these metrics divided into groups and related to their biological role. An additional unsupervised clustering analysis as a **sensitivity analysis** to investigate whether the feature-selection results could be strongly influenced by the original supervised structure of the data has been carried out and included as Supplementary Information S2.

**Figure 5:**
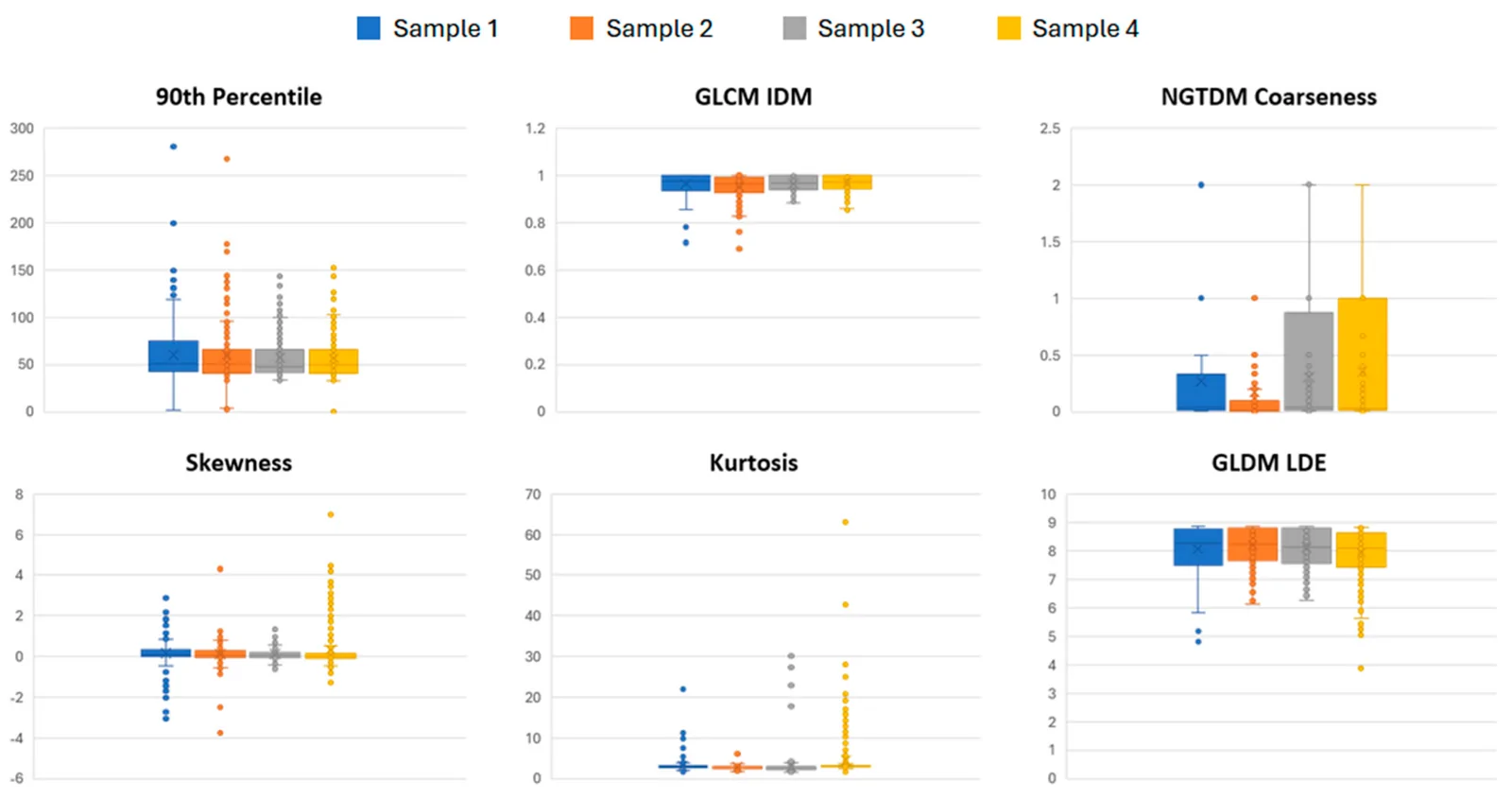
Box and whiskers plots of the robust metrics across the entire data. Each box incorporates 50% of the data (interquartile range). The “x” sign in each box indicates the mean value, the horizontal line in each box indicates the median value. The color dots represent single data points that sit away from the rest of the data (outliers).

Figure 5 presents the statistical representation of the most robust metrics across the entire dataset (including the mean of tissue subtypes). The metrics appear to be homogeneous for all samples with the exception of NGTDM Coarseness.

Table 5 shows the Mean ± SD of the robust metrics, which are proposed as exploratory candidate features as future biomarkers, in each of the tissue subtypes for all the data from 1546 ROIs (in this calculation the outliers observed in Figure 5 were discarded).

**Figure 6:**
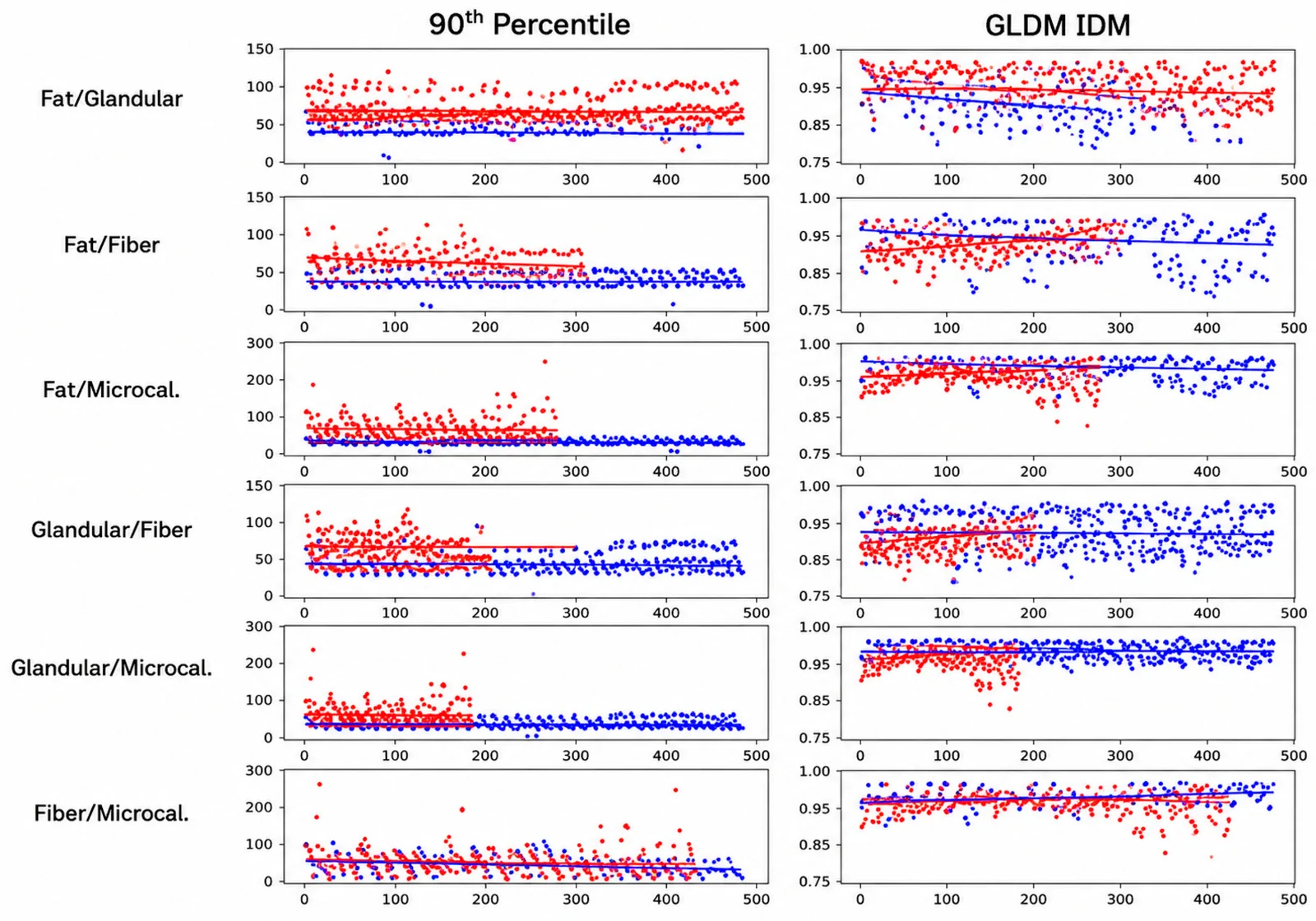
Separation of tissue subtypes from two examples of potential exploratory candidate features, 90th Percentile and GLDM IDM. The red and blue dots represent the actual metric values for each ROI included in the plotted pair of tissue subtypes, while the lines at each boundary separating tissue subtypes correspond to linear fits of the data for each variable. The blue color consistently corresponds to the first subtype in the pair, red color to the second.

As an example, Figure 6 shows the separation of all boundaries based on two of the proposed robust metrics, considering 100% of the data.

**Figure 7:**
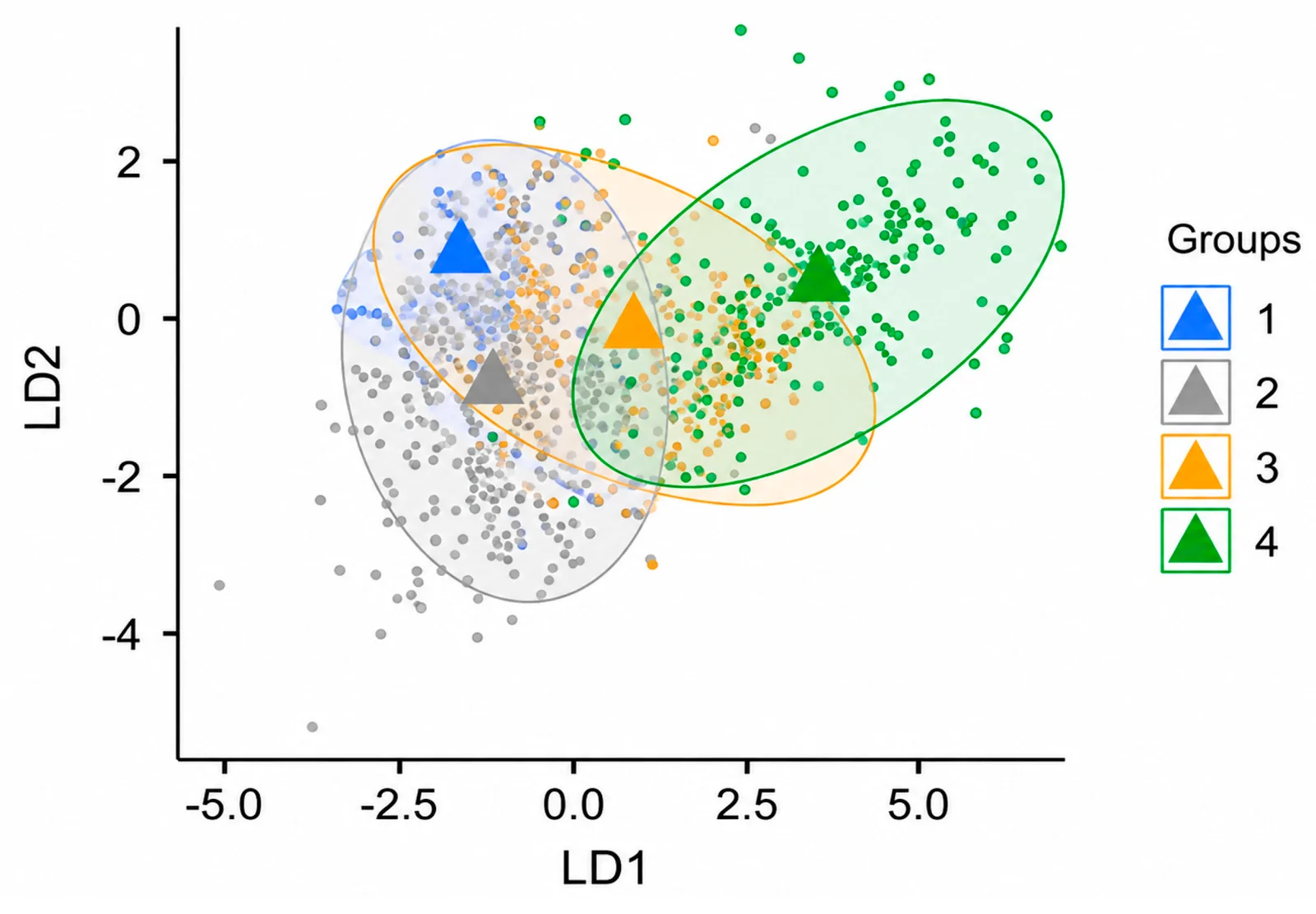
Results of linear discriminant analysis. Groups are: 1 fat—blue, 2 gland—gray, 3 fiber—orange, and 4 microcalcifications—green. LD1 and LD2 are the discriminant functions (linear combinations of the 6 selected variables) that capture the greatest separation between the groups.

Figure 7 shows the LDA results, and Table 6 and Table 7 show the performance metrics. The discriminant analysis reveals four clusters (1 fat—blue, 2 gland—gray, 3 fiber—orange, and 4 microcalcifications—green). The centroids of the clusters, formed from the six robust metrics, are represented by colored triangles. Some overlap between fat and stroma, and between fiber and microcalcifications, is observed within the clusters. In general, fat and stroma are well separated from microcalcifications. There is also some overlap between fiber and stroma. The confusion matrix shows, along its main diagonal, the well-classified pure cases in each cluster and those overlapping in neighboring clusters.

The overall Wilks’ Lambda value was 0.081. That is, only 8% of the total Variance was not explained by the discriminant functions [28]. The significance value was *p* < 0.0001, which indicates that the four clusters are significantly different. To further assess the potential influence of specimen-level dependence, in discriminant analysis, we performed a leave-one-specimen-out cross-validation analysis using speci-men-level separation, in which the classification model was evaluated while keeping spec-imens separated between training and validation subsets. These results appear in Supplementary Information S3.

## 4. Discussion

Given that very small ROIs of only 100 pixels were chosen, it is established as a premise that the ROIs in glandular and fat areas always corresponded, by location, to normal tissue. ROIs over regions where microcalcifications are observed are always considered anomalous, and regions with very bright white fibers, corresponding to the densest fibroglandular tissue, are considered at least doubtful, as they may include fibers where there is tumor infiltration or remnants thereof, according to the results reported by histopathology, which states that there are ducts infiltrated with high-grade carcinoma.

In the following, the radiomic results are interpreted in light of the histopathology and the radiological report for each sample.

The 90th Percentile showed a progressive increase from healthy adipose tissue to abnormal microcalcifications. In normal fat and glandular tissue (stroma), the value was lower than in fibrous tissue (doubtful). The samples were reported as high-grade DCIS, which often generates a desmoplastic reaction (fibrosis) around the ducts, increasing radiological density [29]. Furthermore, two samples showed microcalcifications (abnormalities): the higher value reported for this variable in this tissue subtype is due to the presence of microcalcifications and is suggestive of possible malignancy. In the present study, however, microcalcifications were not classified as malignant or benign due to the small number of samples and the lack of annotated data to corroborate the radiomic result.

The opposite trend is observed in the case of GLCM IDM, which measures local homogeneity. Values close to 1 have been reported for CT in the literature, indicating a very smooth texture [30]. In our analysis, such values have been observed in fat, where the tissue appears rather homogeneous, corresponding to areas of unaffected healthy tissue. In general, slightly lower values have been measured in normal gland (stroma) and fiber, which result in these tissues being marginally more heterogeneous than fat. The value for microcalcifications was the lowest, making this tissue subtype the most heterogeneous. Sample 2, where a high number of microcalcifications is present, had the lowest mean IDM value for microcalcifications at 0.86. On the other hand, samples 1 and 3, where there are areas of chaotic tissue due to the presence of microinvasion foci within the ductal architecture (fiber), yielded lower GLCM IDM values in fiber compared to stromal gland and fat.

Regarding the texture represented by NGTDM Coarseness, the lowest value (indicating the roughest texture) was obtained for microcalcifications, which was very low (close to 0). Similar findings are reported in conventional bCT studies as a sign of malignancy [4,5]; however, future studies are needed to differentiate their typical SR-bCT values for malignant and benign microcalcifications. In the present study, these anomalies were included in several ROIs of the images corresponding to samples 2 and 3. Biologically, this represents macroscopic calcium deposits. However, for sample 3, solid comedonal necrosis is also reported (in histopathology), which consists of dense cellular debris that fills the ducts when high-grade DCIS is present [3]. Comparing the doubtful fiber with the less dense gland, this variable, despite the low values, may suggest that the stroma of the healthy gland was less rough than that of the doubtful fiber. One possible interpretation of this finding is the following: where a desmoplastic reaction is reported as a consequence of the treated tumor bed (samples 1 and 3), the cancer induces the formation of collagen bands to support the tumor [31], and these bands are larger and more uniform structures than the individual glandular acini, which are distorted and fragmented by the invasion of high-grade tumor cells. Finally, fat is recognized as having a less rough texture, even though in high-resolution SR-bCT images, adipose tissue has a very subtle granular appearance. Given the presence of healthy background tissue in these elderly patients (66, 77 years), its structure is the least organized in large patches, compared to the disorder of fibrosis or infiltrating tumors [3].

Positive Skewness, higher in microcalcifications, indicates that most pixels had low values, but there is a long tail towards very bright values. Its high value also in the stroma may suggest an uneven distribution in the tissue density of the samples. Kurtosis was highest in microcalcifications, with values that indicate a distribution with a pronounced peak. Lower Kurtosis values were measured in the other tissue subtypes, including fibers: this could be reasonable considering that the treated tumor bed has components with very similar intensities between the residual tumor or possible post-chemotherapy fibrosis [3,31].

All four samples exhibited outliers in the selected robust metrics, which were excluded from the mean and range of dispersion of the final reported values. The outliers are related to various types of artifacts identified across several images. For example, in sample 1, several slices were affected by star-like artifacts related to the presence of metallic surgical clips. Although attempts were made to locate the ROIs away from this effect, zero impact from this cause cannot be guaranteed. In samples 3 and 4, the data were used only in the largest model, and slices close to the extremity of the sample were affected by some phase-contrast-related artifacts due to the tissue/plastic interface; these effects were more prominent at the lowest energy (25 keV). In all samples, at energies between 35 and 60 keV, some residual uncompensated ring artifacts were visible in a few slices [32].

By reducing variable redundancy and ensuring that the variables selected as significant within linear regression models, capable of separating tissue subtypes fat, gland (stroma), fiber (dense), and microcalcifications, were also robust to variations in sample and energy, a reduced group of only six exploratory candidate features was obtained. This number is considered manageable and interpretable in the medical context.

The results of the general model were subjected to a Benjamini–Hochberg false discovery rate (FDR) correction to account for multiple comparisons (m = 15, α = 0.05) [27]. After the FDR correction, 13 of the 15 metrics remained significant, with the exception of Kurtosis and GLRLM GLNU. It is worth noting that, of the six proposed exploratory candidate features, five were significant, except for Kurtosis, which had a *p*-value of 0.05. Given the exploratory nature of this study and the biological plausibility of Kurtosis as a descriptor of the maximum intensity distributions in microcalcifications, it was maintained as a candidate biomarker pending independent validation.

The most complex tissue separation observed was at the fiber-microcalcification boundary. This is attributed to the greater similarity of both tissue subtypes in terms of histogram, texture, intensity levels, and density [5].

While linear regression provided a practical selection tool for identifying candidate features, the reported classification performance and separability between subtypes are based on linear discriminant analysis, which does not assume homogeneous spacing between tissue categories.

Linear discriminant analysis (LDA) based on the six proposed radiomic features achieved good overall classification accuracy (75%), with excellent identification of healthy fat tissue (96% sensitivity). However, substantial confounding was observed between fibrous tissue and microcalcifications: 19.8% of true microcalcifications were misclassified as fibers, and 8% of true fiber regions were misclassified as microcalcifications or glands (11%). This suggests that while the selected first- and second-order texture metrics capture the overall density and heterogeneity of the tissue, they are insufficient to reliably distinguish highly dense fibrous tissue from microcalcifications. Future work should incorporate shape-based features or higher-order texture metrics specifically optimized for edge detection and focal brightness distributions.

This research represents a first approach to the radiomic characterization of breast tissue using SR-bCT images. Based on the results obtained and those previously reported in the scientific literature for bCT [1,2,3,4,5], several aspects can be hypothesized where the robust metrics obtained in this experiment could be useful in the future and should therefore be studied in depth as soon as sufficient annotated SR-bCT data are available. For example, GLCM-IDM variability could be used to investigate its potential association with tumor malignancy grade. Other possibilities include evaluating the response to chemotherapy, such as by analyzing the reduction in Kurtosis, or using the 90th Percentile to assess its potential utility in differentiating between a solid tumor, a cyst, and an area of necrosis. Finally, the NGTDE Coarseness parameter could be studied to determine whether it plays a significant role in analyzing aggressive tumor growth and predicting prognosis. However, before assessing the feasibility of these applications, it is necessary to study the behavior of these six metrics in the presence of breast masses and to establish a radiomic threshold distinguishing normal dense fibrous tissue from in situ carcinoma.

Currently, radiomics in bCT is being used extensively for various types of classification and prognostics and is integrated with multiple other omics technologies [33]. In the case of imaging, it has been shown that the selected metrics depend heavily on the volume of interest constructed from the ROI, often manually defined [34]. Therefore, there is high variability in the final selection of variables, regardless of whether dimensionality reduction and redundancy techniques are used. Furthermore, the preponderance of shape-based metrics (not included in this work) [33,34,35,36], and texture metrics such as GLRLM and GLDM has been observed.

Other studies [37,38] have emphasized the use of photon counting (PC) detectors and their role in PC-bCT, e.g., for studying breast density [37]. In this last cited study, Skewness was found to be the metric best correlated with the database annotation according to BI-RAD. Its mean value was reported as 0.22 ± 1.0, which corresponds to the 0.2 ± 0.4 reported for fiber in the present study, despite the notable differences in the experimental conditions in both works.

Several limitations of this study should be noted. First, the 1546 ROIs cannot be considered 1546 statistically independent biological observations, since they were obtained from only four mastectomy specimens. They were used to increase the representation of the different tissue types and, particularly, to investigate the stability of radiomic features across energy levels and specimens. Therefore, the linear regression results should be interpreted as exploratory findings requiring validation in a substantially larger number of independent specimens. Second, tissue purity within each region of interest (ROI) was assessed visually only. While the ROIs were designed to be small (100 pixels) and selected to emphasize a single tissue subtype, the actual proportion of reported tissue could vary between approximately 60% and 90%, depending on the region, particularly for microcalcifications (overlapping stroma or fat) and dense fibrous areas (which may contain underlying low-density glandular tissue). ROI purity segmentation and quantification were not performed in this exploratory study. Consequently, the radiomic signature extracted from a given ROI does not exclusively represent the reported tissue subtype, but rather a mixture. This is particularly relevant for second-order texture metrics (GLCM, GLDM, NGTDM), which are sensitive to local intensity transitions between different tissues within the same ROI. Furthermore, because tissue contrast varies with X-ray energy, the relative contribution of each tissue component may change with different energies; for example, some microcalcifications were barely visible at 60 keV. However, the six radiomic candidate features reported here as energy-independent remained significant across the entire energy range despite these mixing effects. A more rigorous approach for future studies would be to quantify the percentage of each tissue component per ROI (e.g., using a simple threshold or semi-automatic segmentation) and include it as a covariate.

On the other hand, the small sample size (four mastectomy samples, all with pathology) limits generalizability. External validation in independent datasets is needed. Also, considering that thousands of radiomic metrics currently exist, it is considered that new experiments should be conducted in the future to find other potential biomarkers suitable for SR-bCT. In particular, the focus should be on achieving better biomarkers to differentiate microcalcifications from dense fibers. Likewise, this analysis should be correlated with observations from conventional hospital bCT.

Shape-related radiomic metrics were not included in this research because none of the samples used contained tumor mass. Therefore, segmentation techniques were not applied to calculate shape-related radiomics. This experiment can be extended to microcalcifications, preferably using automatic segmentation.

Finally, the need to collect sufficient data of this type in the future, with adequate medical notation, is emphasized in order to apply artificial intelligence techniques that, using these or other candidate features, allow classification of the degree of malignancy of masses, microcalcifications and ductal carcinomas in the breast.

## 5. Conclusions

In this experiment, six radiomic metrics, three first-order metrics (90th Percentile, Kurtosis, and Skewness), one second-order texture metric (GLCM IDM), one second-order spatial dependence metric (GLDM Large Dependence Emphasis), and one texture metric (NGTDM Coarseness) separated tissue pairs (microcalcifications/fat, microcalcifications/gland, microcalcifications/fiber, fat/gland, fat/fiber, and gland/fiber) in SR-bCT images.

The selected metrics were independent of the sample and acquisition energy, and they differed significantly between tissue subtypes according to the regression models and discriminant analysis implemented.

It is recommended that these metrics be considered exploratory candidate features as possible biomarkers in future studies to investigate breast tissue abnormalities using bCT and SR-bCT. The specific question of whether to include Kurtosis in this group remains a subject for future studies with larger sample sizes and greater sample variability.

## Figures and Tables

**Table 1:** Radiomic metric values in sample 2.

	Metrics	Mean ± Standard Deviation			
		Fat	Glandule	Fiber	Microc.
1	90th Percentile	48 ± 11	63 ± 17	88 ± 24	108 ± 46
2	Interquartile Range	5 ± 1	11 ± 5	19 ± 8	27 ± 15
3	Median	43 ± 10	52 ± 13	70 ± 19	70 ± 29
4	Robust Mean Absolute Deviation	2.0 ± 0.6	4.0 ± 1.6	7 ± 3	12 ± 8
5	Root Mean Square	55 ± 12	57 ± 13	74 ± 18	73 ± 26
6	Skewness	0.03 ± 0.01	0.4 ± 0.3	−0.1 ± 0.1	0.5 ± 0.8
7	Kurtosis	3 ± 1	3 ± 1	3 ± 2	4 ± 2
8	Entropy	0.2 ± 0.2	0.8 ± 0.3	1 ± 0.4	2 ± 0.6
9	Total Energy × 10^6^	5 ± 2	6 ± 1	10 ± 3	6 ± 5
10	GLCM Cluster Shade	−0.2 ± 0.3	0.4 ± 0.5	−0.1 ± 0.4	3.0 ± 0.5
11	GLCM Correlation	0.4 ± 0.3	0.7 ± 0.1	0.8 ± 0.1	0.8 ± 0.1
12	GLCM Cluster Prominence	0.3 ± 0.5	1.2 ± 1.6	7 ± 8	19 ± 12
13	GLCM Contrast	0.07 ± 0.08	0.12 ± 0.05	0.2 ± 0.1	0.3 ± 0.3
14	GLCM Maximum Probability	0.2 ± 0.2	1 ± 0.3	2 ± 0.3	3 ± 0.6
15	GLCM Difference Entropy	0.4 ± 0.3	0.5 ± 0.5	0.6 ± 0.2	0.9 ± 0.3
16	GLCM Difference Variance	0.03 ± 0.05	0.1 ± 0.1	0.1 ± 0.1	0.2 ± 0.2
17	GLCM IDM	0.95 ± 0.07	0.93 ± 0.03	0.9 ± 0.05	0.86 ± 0.07
18	GLCM IMC1	0.2 ± 0.3	0.4 ± 0.2	0.4 ± 0.2	0.4 ± 0.3
19	GLCM Join Entropy	−0.1 ± 0.1	−0.4 ± 0.1	−0.6 ± 0.1	−0.5 ± 0.1
20	GLCM Sum Entropy	1 ± 0.1	0.7 ± 0.1	0.5 ± 0.2	0.4 ± 0.2
21	GLCM Dependence Non-Uniformity	1390 ± 630	216 ± 144	88 ± 56	31 ± 20
22	GLDM Small Dependence Emphasis	0.1 ± 0.02	0.2 ± 0.02	0.2 ± 0.03	0.2 ± 0.08
23	GLDM Grey Level Non-Uniformity	2227 ± 85	2407 ± 1006	802 ± 382	204 ± 132
24	GLDM Large Dependence Emphasis	8 ± 4	7 ± 2	8 ± 4	7 ± 3
25	GLRLM Grey Level Non uniformity	63 ± 20	206 ± 82	79 ± 45	31 ± 12
26	GLRLM Grey Level Variance	0.18 ± 0.09	0.28 ± 0.07	0.5 ± 0.2	0.8 ± 0.7
27	GLRLM Long Run Emphasis	74 ± 18	124 ± 45	48 ± 12	14 ± 6
28	GLRLM Run Entropy	−0.004 ± 0.012	1.2 ± 0.4	1.9 ± 0.4	2 ± 0.7
29	GLRLM Run Length Non-Uniformity	31 ± 10	48 ± 29	20 ± 10	20 ± 19
30	GLRLM Short Run Emphasis	9 ± 0.1	8 ± 0.4	7 ± 0.4	6 ± 1
31	NGTDM Busyness	2472 ± 59	2655 ± 1133	696 ± 306	184 ± 120
32	NGTDM Coarseness	0.2 ± 0.3	0.1 ± 0.05	0.2 ± 0.2	0.06 ± 0.01
33	NGTDM Complexity	0.03 ± 0.04	0.2 ± 0.2	0.6 ± 0.6	2.4 ± 2
34	NGTDM Contrast	0.2 ± 0.2	0.02 ± 0.02	0.05 ± 0.08	0.40 ± 1

**Table 2:** Significant metrics in each of the seven models. An “x” indicates that the corresponding metric met the significance criterion in that model.

Metrics with Significance for Separating Tissue Subtypes	Sample 1 at 25 keV	1 at 60 keV	1 at Five Energies	Sample 2 at 25 keV	2 at 60 keV	2 at Five Energies	Four Samples at Five Energies
90th Percentile			x	x	x	x	x
Interquartile Range							
Median		x	x	x			
Robust Mean Absolute Deviation							
Skewness		x	x	x	x	x	x
Kurtosis		x	x	x	x		x
Root Mean Square			x	x	x	x	
Total Energy			x				x
Entropy			x				
GLCM Cluster Shade							
GLCM Cluster Prominence							
GLCM Correlation					x		x
GLCM Contrast							
GLCM Maximum Probability							x
GLCM Difference Entropy	x	x					
GLCM Difference Variance	x			x			
GLCM IDM			x	x	x	x	x
GLCM IMC1							x
GLCM Join Entropy				x	x	x	x
GLCM Sum Entropy				x	x		x
GLCM Dependence Non-Uniformity							
GLDM Small Dependence Emphasis						x	x
GLDM Grey Level Non-Uniformity							x
GLDM Large Dependence Emphasis			x	x	x		x
GLRLM Grey Level Non-Uniformity				x		x	x
GLRLM Grey Level Variance				x		x	
GLRLM Long Run Emphasis	x					x	
GLRLM Run Entropy							
GLRLM Run Length Non-Uniformity		x					
GLRLM Short Run Emphasis	x	x					
NGTDM Busyness				x	x	x	
NGTDM Coarseness	x		x		x		x
NGTDM Complexity				x			
NGTDM Contrast				x	x		

**Table 3:** Significant metrics in the general model.

Model 7, R = 0.816, 1546 ROIs, All Samples, All Energies	
Metric	*p*-Value
90th Percentile	<0.001
Kurtosis	0.05
Skewness	0.001
Total Energy	0.002
GLCM Correlation	0.04
GLCM IDM	0.039
GLCM ImC1	0.027
GLRLM Grey Level Non-Uniformity	0.05
GLCM Maximum Probability	0.001
GLCM Sum Entropy	0.002
GLDM Large Dependence Emphasis	0.045
GLDM Small Dependence Emphasis	0.039
NGTDM Coarseness	0.027
GLCM Join Entropy	0.030
GLDM Grey Level Non-Uniformity	0.038

**Table 4:** Robust metrics with their biological roles.

Metric Family	Metric	Biological Role
First order	90th Percentile	It captured peak densities, clearly identifying each subtype, particularly microcalcifications compared to the other subtypes.
	Kurtosis	A sharp asymmetry distinguished microcalcifications from fat and glandular tissue.
	Skewness	Histogram asymmetry differentiated fat from the other, denser tissue subtypes.
Second-order GLCM Texture	IDM	It measured homogeneity, distinguishing homogeneous fat and stroma vs. heterogeneous fibrous tissues or microcalcifications.
Second-order GLDM spatial dependence	LDE	It distinguished large homogeneous dependent areas: fat, connected fibers and stroma vs. microcalcifications.
Second-order NGTDM Texture	Coarseness	Overall, granularity was very low for fat and higher for microcalcifications.

**Table 5:** Values of the metrics proposed as exploratory candidate features in tissue subtypes.

Metric	Fat	Gland	Fiber	Microcalcification
Skewness	0.04 ± 0.05	0.8 ± 0.3	0.2 ± 0.4	0.7 ± 0.8
Kurtosis	3 ± 0.2	3 ± 0.4	3 ± 2	13 ± 7
90th Percentile	44 ± 13	59 ± 17	78 ± 24	88 ± 1
GLCM IDM	1 ± 0.03	0.95 ± 0.02	0.94 ± 0.02	0.91 ± 0.04
GLDM LDE	8 ± 4	8 ± 2	8 ± 1	7 ± 2
NGTDM Coarseness	0.5 ± 0.8	0.2 ± 0.1	0.2 ± 0.1	0.1 ± 0.012

**Table 6:** Confusion matrix.

	Predicted Tissue Subtypes			
Real Tissue Subtypes	Fat	Gland	Fiber	Microcalcifications
Fat	532	23	0	0
Gland	150	367	31	3
Fiber	64	26	129	19
Microcalcifications	9	10	40	143

**Table 7:** Performance of metrics by class. (**a**) Global metrics. (**b**) Metrics by tissue subtype.

(**a**)				
**Metric**			**Overall**	**95% CI**
Accuracy			75.74%	73.61–77.88%
Balanced Accuracy			71.86%	69.40–74.32%
Precision (Macro-average)			76.95%	74.52–79.32%
Recall/Sensitivity (Macro-average)			71.86%	69.40–74.32%
Specificity (Macro-average)			91.13%	90.38–91.87%
F1-score (Macro-average)			73.30%	70.80–75.72%
(**b**)				
**Tissue Subtype**	**Precision (%)**	**Recall/** **Sensitivity (%)**	**Specificity (%)**	**F1-Score (%)**
Fat	70.46	95.86	77.50	81.22
Gland	86.15	66.61	94.07	75.13
Fiber	64.50	54.20	94.57	58.90
Microcalcific.	86.67	70.79	98.36	77.93
Macro-average	76.95	71.86	91.13	73.30

Note: In total, 1546 ROIs were included. Accuracy was calculated as the proportion of correctly classified ROIs. Balanced accuracy, precision, recall, specificity, and F1-score were calculated as macro-averages across the four tissue subtypes. The 95% confidence intervals were estimated via bootstrap resampling.

## Data Availability

The datasets used in this article are not publicly available because they are part of an ongoing study involving sensitive patient data. These data were only authorized for the purpose of this research, under an ethical protocol, and with the informed consent of the individuals involved. The anonymity of the sources is guaranteed.

## Supplementary Materials

The following supporting information can be downloaded at: https://www.mdpi.com/article/10.3390/tomography12090124/s1, S1: Tables showing the significant variables for each of the six partial linear regression models; S2: Clustering analysis tables with Games–Howell post-hoc comparisons for one specimen at all energy levels, the complete dataset (four specimens at all energy levels), and one specimen at 25 keV; S3: Tables from Cross validation analysis in Linear Discriminant Analysis.
