# Supplementary material for: Radiomic Characterization of Breast Tissue from Breast CT Images Obtained with Synchrotron Beams
Source: Tomography. 2026 Aug 29;12(9):124. doi: 10.3390/tomography12090124 (PMC13611729; doi:10.3390/tomography12090124)
Supplement: Supplementary file 1 [file tomography-12-00124-s001.zip › S1 Partial models.pdf]

Partial Models

Model 1, R=0.907, 73 ROI, Sample 1 at 25 keV	
**Metric**	**Signification p**
GLCM Difference Entropy	0.007
GLCM Difference Variance	0.004
GLRLM Long Run Emphasis	0.04
GLRLM Short Run Emphasis	0.033
NGTDM Coarseness	0.004
**Model 2, R=0.976, 73 ROI, Sample 1 at 60 keV**	
**Metric**	**Signification p**
Kurtosis	0.023
Skeweness	0.017
GLRML Run Lenght Non Uniformity	0.023
NGTDM Buyness	0.05
Median	0.08
GLCM Difference Entropy	0.076
**Model 3, R=0.995, 94 ROI, Sample 2 at 25 keV**	
**Metric**	**Signification p**
Percentil 90	0.014
Kurtosis	< 0.001
Median	0.029
Root Mean Square	0.001
Skeweness	0.014
GLDM Difference Variance	0.004
GLCM IDM	0.05
GLCM Join Entropy	0.038
GLCM Sum Entropy	0.021
GLRML Grey Level Non Uniformity	0.009
GLRML Grey Level Variance	0.001
GLDM Large Dependence Emphasis	0.018
NGTDM Buyness	0.006
NGTDM complexity	0.001
NGTDM Contrast	0.001

Model 4, R=0.989, 94 ROI, Sample 2 at 60 keV	
**Metric**	**Signification p**
Kurtosis	0.005
Root Mean Square	0.05
GLDM Small Dependence Emphasis	0.011
Percentil 90	0.1
Skeweness	0.1
Correlation	0.1
GLCM IDM	0.07
GLCM Join entropy	0.1
GLCM Sum entropy	0.1
GLDM Large Dependence Emphasis	0.1
NGTD Buyness	0.1
NGTDM Coarsness	0.08
NGTDM Contrast	0.07
**Model 5, R=0.907, 413 ROI, Sample 1, all energies**	
**Metric**	**Signification p**
Percentil 90	0.001
Entropy	0.019
Kurtosis	0.034
Median	< 0.001
Root Mean Square	0.001
Skeweness	< 0.001
Total Energy	0.005
GLDM IDM	0.011
GLDM Large Dependence Emphasis	0.001
NGTDM Coarsness	0.04
**Model 6, R=0.812, 378 ROI, Sample 2, all energies**	
**Metric**	**Signification p**
Entropy	<0.001
Skeweness	0.05
GLCM IDM	0.001
GLCM Join Entropy	0.002
GLRML Grey Level Non Uniformity	0.004
GLRML Short Run Emphasis	0.004
GLDM Small dependence Enphasis	0.027
Percentil 90	0.07

Root Mean Square	0.06
GLRML Grey Level variance	0.07
GLRML Large Run Emphasis	0.08
