# Supplementary material for: Radiomic Characterization of Breast Tissue from Breast CT Images Obtained with Synchrotron Beams
Source: Tomography. 2026 Aug 29;12(9):124. doi: 10.3390/tomography12090124 (PMC13611729; doi:10.3390/tomography12090124)
Supplement: Supplementary file 1 [file tomography-12-00124-s001.zip › S2 Sensitivity analysis Supplementary.pdf]

Sensitivity analysis.

We performed an unsupervised clustering analysis as a **sensitivity analysis** to investigate whether the feature-selection results could be strongly influenced by the original supervised structure of the data.

This analysis was first performed on specimen 2 (patient 2), because this was the only specimen containing all four tissue subtypes with a sufficient number of ROIs corresponding to microcalcifications. Four clusters were generated without imposing correspondence between the clusters and the predefined tissue labels. Subsequently, one-way ANOVA (as the reviewer suggests) was used to identify radiomic features that differed significantly among the four clusters. Because the Levene test indicated non-homogeneity of variance for the variables, Games–Howell post-hoc comparisons were also performed.

The unsupervised clustering approach identified 19 of the 34 radiomic features as significantly different among the four clusters, with most showing $p < 0.001$. Thus, this approach was considerably more inclusive than the original feature-selection strategy. In the original workflow, the Sample 2 five-energy model identified 12 significant features, and 10 of these were also identified by this clustering analysis. The two candidate features from the original analysis that were not identified in the clustering analysis were kurtosis and skewness.

To further evaluate the consistency of this observation, the same clustering procedure was subsequently applied to the complete dataset. Again, 18 of the 34 features showed significant differences among the four clusters. The intersection between the features identified in specimen 2 and those identified in the complete dataset comprised 17 features. Importantly, four of the six candidate radiomic features identified by our original workflow were included among these 17 common features.

These results indicate that the unsupervised clustering approach taking only the specimen as a grouping variable provides a consistent but substantially less parsimonious feature-selection strategy. It identifies a larger set of potentially discriminative features and therefore represents a more inclusive approach, with a lower risk of excluding potentially relevant variables at this exploratory stage. However, this greater inclusiveness does not by itself provide a mechanism for distinguishing noise, potentially redundant or condition-specific features from the most robust candidates.

Then, as an extra verification step, an analysis was conducted using just the 25 keV energy level for sample 2; once again, 19 variables were included, showing a high degree of agreement with the previous analysis. It is concluded that this approach does not allow for the reduction of noise or potential remaining redundancies. It is substantially less parsimonious than the original feature-selection strategy. The clustering approach produced a similarly large number of significant variables regardless of the energy level or sample considered and therefore did not provide an effective mechanism for feature reduction.

The following tables are cut and pasted directly from the Jamovi output. The variable legend is as follows:

E. Percentile 90, I Kurtosis, G Entropy, H interquatile range, M Median, P Mean robust absolute

deviation, Q Mean square root, R Skewness, S Total Energy, X GLCM Cluster Shade, Z GLCM Contrast, AA GLCM Correlation, AC GLCM Difference Entropy, AF GLCM IDM, AI GLCM IMC1, AN GLCM Join Entropy , AR GLCM Sum Entropy, AT GLRLM GLNU, AV GLRLM Grey Level Variance, AX GLRLM Long Run Emphasis, BB GLRLM Run Entropy, BC GLRLM RLNU, BG GLRLM Short Run Emphasis CA GLDM Dependence Non Uniformity, CG GLDM LDE, CD GLDM GLNU, CK GLDM Small Dependence Emphasis, CN NGTDM Buyness, CO NGTDM Coarsness, CP NGTDM Complexity, CQ NGTDM Contrast, CR NGTDM Strength, W GLCM Cluster Prominance, AD GLCM Cluster Variance

Clustering sample 2

ANOVA One Factor (Fisher)

Variables	F	gl1	gl2	p
E	82.90	3	409	<.001
I	3.64	3	409	.013
G	119.19	3	409	<.001
H	100.69	3	409	<.001
M	59.87	3	409	<.001
P	94.89	3	409	<.001
Q	67.38	3	409	<.001
R	33.14	3	409	<.001
S	27.81	3	409	<.001
X	3.88	3	409	.009
Z	43.88	3	409	<.001
AA	13.60	3	409	<.001
AC	70.70	3	409	<.001
AF	69.28	3	409	<.001
AI	129.40	3	409	<.001
AN	111.93	3	409	<.001
AR	115.52	3	409	<.001
AT	55.82	3	409	<.001
AV	11.82	3	409	<.001
AX	56.13	3	409	<.001
BB	39.38	3	409	<.001
BC	27.15	3	409	<.001
BG	16.10	3	409	<.001
CA	215.84	3	409	<.001
CD	214.76	3	409	<.001

CG	103.34	3	409	<.001
CK	64.52	3	409	<.001
CN	7.41	3	409	<.001
CO	23.88	3	409	<.001
CP	7.19	3	409	<.001
CQ	21.32	3	409	<.001
CR	4.92	3	409	.002
W	3.19	3	409	.024
AD	50.47	3	409	<.001

Test post hoc

Test Post-Hoc - Games-Howell – E

	Clusters	1	2	3	4
1	Difference of means	—	-12.6	-37.1	-49.7
	p-valor	—	<.001	<.001	<.001
2	Difference of means		—	-24.5	-37.1
	p-valor		—	<.001	<.001
3	Difference of means			—	-12.7
	p-valor			—	.298
4	Difference of means				—
	p-valor				—

Test Post-Hoc - Games-Howell – I

		1	2	3	4
1	Difference of means	—	0.275	-0.336	-0.2649
	p-valor	—	.195	.522	.742
2	Difference of means		—	-0.611	-0.5402
	p-valor		—	.129	.258
3	Difference of means			—	0.0711
	p-valor			—	.997
4	Difference of means				—
	p-valor				—

rTest Post-Hoc - Games-Howell – G

		1	2	3	4
1	Difference of means	—	-0.445	-0.902	-1.202
	p-valor	—	<.001	<.001	<.001
2	Difference of means		—	-0.457	-0.758
	p-valor		—	<.001	<.001
3	Difference of means			—	-0.301
	p-valor			—	.079
4	Difference of means				—
	p-valor				—

Test Post-Hoc - Games-Howell – H

		1	2	3	4
1	Difference of means	—	-5.02	-12.26	-17.29
	p-valor	—	<.001	<.001	<.001
2	Difference of means		—	-7.23	-12.26
	p-valor		—	<.001	<.001
3	Difference of means			—	-5.03
	p-valor			—	.160
4	Difference of means				—
	p-valor				—

Test Post-Hoc - Games-Howell – M

		1	2	3	4
1	Difference of means	—	-7.34	-26.0	-24.95
	p-valor	—	<.001	<.001	<.001
2	Difference of means		—	-18.7	-17.61
	p-valor		—	<.001	<.001
3	Difference of means			—	1.10
	p-valor			—	.994
4	Difference of means				—
	p-valor				—

Test Post-Hoc - Games-Howell – P

		1	2	3	4
1	Difference of means	—	-2.04	-5.05	-7.48
	p-valor	—	<.001	<.001	<.001
2	Difference of means		—	-3.00	-5.44
	p-valor		—	<.001	<.001
3	Difference of means			—	-2.44
	p-valor			—	.126
4	Difference of means				—
	p-valor				—

Test Post-Hoc - Games-Howell – Q

		1	2	3	4
1	Difference of means	—	-8.20	-26.9	-31.30
	p-valor	—	<.001	<.001	<.001
2	Difference of means		—	-18.7	-23.11
	p-valor		—	<.001	<.001
3	Difference of means			—	-4.36
	p-valor			—	.806
4	Difference of means				—
	p-valor				—

Test Post-Hoc - Games-Howell – R

		1	2	3	4
1	Difference of means	—	-0.241	0.136	-0.569
	p-valor	—	<.001	.384	<.001
2	Difference of means		—	0.377	-0.328
	p-valor		—	<.001	.012
3	Difference of means			—	-0.705
	p-valor			—	<.001
4	Difference of means				—
	p-valor				—

Test Post-Hoc - Games-Howell – S

		1	2	3	4

		1	2	3	4
1	Difference of means	—	-3.79e+6	-1.10e+6	1.37e+6
	p-valor	—	<.001	.229	.058
2	Difference of means		—	2.69e+6	5.16e+6
	p-valor		—	.001	<.001
3	Difference of means			—	2.47e+6
	p-valor			—	.006
4	Difference of means				—
	p-valor				—

Test Post-Hoc - Games-Howell – X

		1	2	3	4
1	Difference of means	—	-0.291	0.196	-39.8
	p-valor	—	<.001	.666	.628
2	Difference of means		—	0.487	-39.5
	p-valor		—	.032	.633
3	Difference of means			—	-40.0
	p-valor			—	.624
4	Diferencia de medias				—
	p-valor				—

Test Post-Hoc - Games-Howell – Z

		1	2	3	4
1	Difference of means	—	-0.0473	-0.0897	-0.1783
	p-valor	—	<.001	<.001	<.001
2	Difference of means		—	-0.0425	-0.1311
	p-valor		—	<.001	.003
3	Difference of means			—	-0.0886
	p-valor			—	.075
4	Difference of means				—
	p-valor				—

Test Post-Hoc - Games-Howell – AA

		1	2	3	4
1	Difference of means	—	-0.0810	-0.228	-0.2976
	p-valor	—	.214	<.001	<.001
2	Difference of means		—	-0.147	-0.2166
	p-valor		—	.001	<.001
3	Difference of means			—	-0.0692
	p-valor			—	.329
4	Difference of means				—
	p-valor				—

Test Post-Hoc - Games-Howell – AC

		1	2	3	4
1	Difference of means	—	-0.204	-0.345	-0.508
	p-valor	—	<.001	<.001	<.001
2	Difference of means		—	-0.141	-0.304
	p-valor		—	<.001	<.001
3	Difference of means			—	-0.163
	p-valor			—	.014
4	Difference of means				—
	p-valor				—

Test Post-Hoc - Games-Howell – AF

		1	2	3	4
1	Difference of means	—	0.0236	0.0440	0.0752
	p-valor	—	<.001	<.001	<.001
2	Difference of means		—	0.0204	0.0516

	p-valor		—	<.001	<.001
3	Difference of means			—	0.0312
	p-valor			—	.004
4	Difference of means				—
	p-valor				—

Test Post-Hoc - Games-Howell – AI

		1	2	3	4
1	Difference of means	—	0.245	0.366	0.367
	p-valor	—	<.001	<.001	<.001
2	Difference of means		—	0.122	0.122
	p-valor		—	<.001	<.001
3	Difference of means			—	8.50e-4
	p-valor			—	1.000
4	Difference of means				—
	p-valor				—

Test Post-Hoc - Games-Howell – AN

		1	2	3	4
1	Difference of means	—	-0.667	-1.294	-1.795
	p-valor	—	<.001	<.001	<.001
2	Difference of means		—	-0.626	-1.128
	p-valor		—	<.001	<.001
3	Difference of means			—	-0.501
	p-valor			—	.032
4	Difference of means				—
	p-valor				—

Test Post-Hoc - Games-Howell – AR

		1	2	3	4
1	Difference of means	—	-0.620	-1.205	-1.638
	p-valor	—	<.001	<.001	<.001
2	Difference of means		—	-0.585	-1.018
	p-valor		—	<.001	<.001

3	Difference of means			—	-0.433
	p-valor			—	.043
4	Difference of means				—
	p-valor				—

Test Post-Hoc - Games-Howell – AT

		1	2	3	4
1	Difference of means	—	-76.5	13.5	40.9
	p-valor	—	<.001	.142	<.001
2	Difference of means		—	90.0	117.4
	p-valor		—	<.001	<.001
3	Difference of means			—	27.4
	p-valor			—	<.001
4	Difference of means				—
	p-valor				—

Test Post-Hoc - Games-Howell – AV

		1	2	3	4
1	Difference of means	—	-0.109	-0.350	-1.313
	p-valor	—	<.001	<.001	.140
2	Difference of means		—	-0.242	-1.204
	p-valor		—	<.001	.198
3	Difference of means			—	-0.962
	p-valor			—	.385
4	Difference of means				—
	p-valor				—

Test Post-Hoc - Games-Howell – AX

		1	2	3	4
1	Difference of means	—	719	1402	1579
	p-valor	—	<.001	<.001	<.001
2	Difference of means		—	683	860
	p-valor		—	<.001	<.001
3	Difference of means			—	177

	p-valor	—	.004
4	Difference of means		—
	p-valor		—

Test Post-Hoc - Games-Howell – BB

		1	2	3	4
1	Difference of means	—	-1.71	-2.165	-2.046
	p-valor	—	<.001	<.001	<.001
2	Difference of means		—	-0.458	-0.339
	p-valor		—	.214	.463
3	Difference of means			—	0.119
	p-valor			—	.966
4	Difference of means				—
	p-valor				—

Test Post-Hoc - Games-Howell – BC

		1	2	3	4
1	Difference of means	—	-4.89	17.8	20.07
	p-valor	—	.245	<.001	<.001
2	Difference of means		—	22.6	24.96
	p-valor		—	<.001	<.001
3	Difference of means			—	2.31
	p-valor			—	.939
4	Difference of means				—
	p-valor				—

Test Post-Hoc - Games-Howell – BG

		1	2	3	4
1	Difference of means	—	-0.0663	-0.1029	-0.1195
	p-valor	—	<.001	<.001	<.001
2	Difference of means		—	-0.0366	-0.0532
	p-valor		—	.152	.072

		1	2	3	4
3	Difference of means			—	-0.0166
	p-valor			—	.900
4	Difference of means				—
	p-valor				—

Test Post-Hoc - Games-Howell – CA

		1	2	3	4
1	Difference of means	—	-242	1434	1863
	p-valor	—	.019	<.001	<.001
2	Difference of means		—	1676	2105
	p-valor		—	<.001	<.001
3	Difference of means			—	429
	p-valor			—	<.001
4	Difference of means				—
	p-valor				—

Test Post-Hoc - Games-Howell – CD

		1	2	3	4
1	Difference of means	—	-28.9	1751	2138
	p-valor	—	.989	<.001	<.001
2	Difference of means		—	1780	2167
	p-valor		—	<.001	<.001
3	Difference of means			—	386
	p-valor			—	<.001
4	Difference of means				—
	p-valor				—

Test Post-Hoc - Games-Howell – CG

		1	2	3	4
1	Difference of means	—	0.444	0.929	1.638
	p-valor	—	<.001	<.001	<.001

2	Difference of means		—	0.484	1.193
	p-valor		—	<.001	<.001
3	Difference of means			—	0.709
	p-valor			—	<.001
4	Difference of means				—
	p-valor				—

Test Post-Hoc - Games-Howell – CO

		1	2	3	4
1	Difference of means	—	-0.0187	-0.0412	-0.0722
	p-valor	—	<.001	<.001	<.001
2	Difference of means		—	-0.0226	-0.0535
	p-valor		—	<.001	<.001
3	Difference of means			—	-0.0309
	p-valor			—	.009
4	Difference of means				—
	p-valor				—

Test Post-Hoc - Games-Howell – CN

		1	2	3	4
1	Difference of means	—	-174	8.21	7.844
	p-valor	—	.003	.276	.423
2	Difference of means		—	182.56	182.202
	p-valor		—	.002	.002
3	Difference of means			—	-0.363
	p-valor			—	.999
4	Difference of means				—

	p-valor				—

Test Post-Hoc - Games-Howell – CK

		1	2	3	4
1	Difference of means	—	0.310	0.3324	0.3586
	p-valor	—	<.001	<.001	<.001
2	Difference of means		—	0.0228	0.0489
	p-valor		—	.968	.683
3	Difference of means			—	0.0262
	p-valor			—	.965
4	Difference of means				—
	p-valor				—

Test Post-Hoc - Games-Howell – CP

		1	2	3	4
1	Difference of means	—	-0.178	-0.716	-3.95
	p-valor	—	<.001	<.001	.339
2	Difference of means		—	-0.538	-3.77
	p-valor		—	<.001	.379
3	Difference of means			—	-3.23
	p-valor			—	.515
4	Difference of means				—
	p-valor				—

Test Post-Hoc - Games-Howell – CQ

		1	2	3	4
1	Difference of means	—	-0.00631	-0.00580	-0.01339
	p-valor	—	<.001	<.001	<.001
2	Difference of means		—	5.11e-4	-0.00708
	p-valor		—	.972	.002
3	Difference of means			—	-0.00759
	p-valor			—	<.001

4	Difference of means				—
	p-valor				

2

Test Post-Hoc - Games-Howell – CR

		1	2	3	4
1	Difference of means	—	0.0194	-0.0532	-1.10
	p-valor	—	.913	.529	.530
2	Difference of means		—	-0.0726	-1.12
	p-valor		—	.221	.515
3	Difference of means			—	-1.05
	p-valor			—	.571
4	Difference of means				—
	p-valor				—

Test Post-Hoc - Games-Howell – W

		1	2	3	4
1	Difference of means	—	-0.859	-6.35	-1135
	p-valor	—	<.001	<.001	.698
2	Difference of means		—	-5.49	-1134
	p-valor		—	<.001	.698
3	Difference of means			—	-1129
	p-valor			—	.701
4	Difference of means				—
	p-valor				—

Test Post-Hoc - Games-Howell – AD

		1	2	3	4
1	Difference of means	—	-0.0417	-0.0751	-0.1343
	p-valor	—	<.001	<.001	<.001
2	Difference of means		—	-0.0334	-0.0926
	p-valor		—	<.001	.001
3	Difference of means			—	-0.0591

	p-valor	—	.074
4	Difference of means		—
	p-valor		—

Clustering Whole Sample

ANOVA one Factor (Fisher)

	F	gl1	gl2	p
E	263.068	3	1542	<.001
G	293.921	3	1542	<.001
H	285.067	3	1542	<.001
I	35.371	3	1542	<.001
M	174.963	3	1542	<.001
P	286.907	3	1542	<.001
Q	207.569	3	1542	<.001
R	198.989	3	1542	<.001
S	48.722	3	1542	<.001
W	5.665	3	1542	<.001
X	10.909	3	1542	<.001
AA	33.348	3	1542	<.001
AC	129.278	3	1542	<.001
AD	113.786	3	1542	<.001
AF	110.991	3	1542	<.001
AI	394.019	3	1542	<.001
AN	260.550	3	1542	<.001
AR	274.468	3	1542	<.001
AT	87.813	3	1542	<.001
AV	0.976	3	1542	.403
AX	170.791	3	1542	<.001
BB	6.831	3	1542	<.001
BC	96.289	3	1542	<.001
BG	0.406	3	1542	.749
CA	417.632	3	1542	<.001
CD	471.076	3	1542	<.001
CG	3.867	3	1542	.009

CK	0.538	3	1542	.656
CN	7.167	3	1542	<.001
CO	37.938	3	1542	<.001
CP	5.826	3	1542	<.001
CQ	0.581	3	1542	.627
CR	0.836	3	1542	.474

Test post hoc

Test Post-Hoc Games-Howell – E

		1	2	3	4
1	Difference of means	—	-11.9	-31.0	-41.7
	p-valor	—	<.001	<.001	<.001
2	Difference of means		—	-19.1	-29.9
	p-valor		—	<.001	<.001
3	Difference of means			—	-10.8
	p-valor			—	.003
4	Difference of means				—
	p-valor				—

Test Post-Hoc Games-Howell – G

		1	2	3	4
1	Difference of means	—	-0.383	-0.655	-0.995
	p-valor	—	<.001	<.001	<.001
2	Difference of means		—	-0.272	-0.612
	p-valor		—	<.001	<.001
3	Difference of means			—	-0.340
	p-valor			—	<.001
4	Difference of means				—
	p-valor				—

Test Post-Hoc Games-Howell – H

		1	2	3	4
1	Difference of means	—	-4.59	-9.91	-13.76
	p-valor	—	<.001	<.001	<.001
2	Difference of means		—	-5.33	-9.17

	p-valor		—	<.001	<.001
3	Difference of means			—	-3.84
	p-valor			—	.002
4	Difference of means				—
	p-valor				—

Test Post-Hoc Games-Howell – I

		1	2	3	4
1	Difference of means	—	0.220	-0.369	-2.26
	p-valor	—	.118	.639	<.001
2	Difference of means		—	-0.589	-2.48
	p-valor		—	.275	<.001
3	Difference of means			—	-1.89
	p-valor			—	<.001
4	Difference of means				—
	p-valor				—

Test Post-Hoc Games-Howell – M

		1	2	3	4
1	Difference of means	—	-8.13	-21.6	-20.963
	p-valor	—	<.001	<.001	<.001
2	Difference of means		—	-13.5	-12.832
	p-valor		—	<.001	<.001
3	Difference of means			—	0.646
	p-valor			—	.986
4	Difference of means				—
	p-valor				—

Test Post-Hoc Games-Howell – P

		1	2	3	4
1	Difference of means	—	-1.86	-4.08	-6.03
	p-valor	—	<.001	<.001	<.001
2	Difference of means		—	-2.22	-4.17
	p-valor		—	<.001	<.001
3	Difference of means			—	-1.95
	p-valor			—	<.001
4	Difference of means				—

	p-valor	—

Test Post-Hoc Games-Howell – Q

		1	2	3	4
1	Difference of means	—	-8.36	-22.7	-26.57
	p-valor	—	<.001	<.001	<.001
2	Difference of means		—	-14.3	-18.21
	p-valor		—	<.001	<.001
3	Difference of means			—	-3.91
	p-valor			—	.260
4	Difference of means				—
	p-valor				—

Test Post-Hoc Games-Howell – R

		1	2	3	4
1	Difference of means	—	-0.00673	-0.0718	-1.047
	p-valor	—	.989	.384	<.001
2	Difference of means		—	-0.0651	-1.040
	p-valor		—	.554	<.001
3	Difference of means			—	-0.975
	p-valor			—	<.001
4	Difference of means				—
	p-valor				—

Test Post-Hoc Games-Howell – S

		1	2	3	4
1	Difference of means	—	-2.52e+6	-2.13e+6	-8451
	p-valor	—	<.001	<.001	1.000
2	Difference of means		—	391902	2.51e+6
	p-valor		—	.700	<.001
3	Difference of means			—	2.12e+6
	p-valor			—	<.001
4	Difference of means				—
	p-valor				—

Test Post-Hoc Games-Howell – W

		1	2	3	4
1	Difference of means	—	-0.598	-5.16	-388
	p-valor	—	<.001	<.001	.380
2	Difference of means		—	-4.56	-388
	p-valor		—	<.001	.382
3	Difference of means			—	-383
	p-valor			—	.392
4	Difference of means				—
	p-valor				—

Test Post-Hoc Games-Howell – X

		1	2	3	4
1	Difference of means	—	-0.174	-0.2172	-17.9
	p-valor	—	<.001	.038	.119
2	Difference of means		—	-0.0433	-17.7
	p-valor		—	.952	.125
3	Difference of means			—	-17.7
	p-valor			—	.126
4	Difference of means				—
	p-valor				—

Test Post-Hoc Games-Howell – AA

		1	2	3	4
1	Difference of means	—	-0.0448	-0.1260	-0.259
	p-valor	—	.157	<.001	<.001
2	Difference of means		—	-0.0812	-0.214
	p-valor		—	.003	<.001
3	Difference of means			—	-0.133
	p-valor			—	<.001
4	Difference of means				—
	p-valor				—

Test Post-Hoc Games-Howell – AC

		1	2	3	4
1	Difference of means	—	-0.162	-0.2175	-0.378
	p-valor	—	<.001	<.001	<.001
2	Difference of means		—	-0.0551	-0.216

		1	2	3	4
	p-valor		—	.011	<.001
3	Difference of means			—	-0.161
	p-valor			—	<.001
4	Difference of means				—
	p-valor				—

Test Post-Hoc Games-Howell – AD

		1	2	3	4
1	Difference of means	—	-0.0312	-0.0433	-0.0952
	p-valor	—	<.001	<.001	<.001
2	Difference of means		—	-0.0121	-0.0640
	p-valor		—	.012	<.001
3	Difference of means			—	-0.0519
	p-valor			—	<.001
4	Difference of means				—
	p-valor				—

Test Post-Hoc Games-Howell – AF

		1	2	3	4
1	Difference of means	—	0.0169	0.02391	0.0517
	p-valor	—	<.001	<.001	<.001
2	Difference of means		—	0.00696	0.0347
	p-valor		—	<.001	<.001
3	Difference of means			—	0.0278
	p-valor			—	<.001
4	Difference of means				—
	p-valor				—

Test Post-Hoc Games-Howell – AI

		1	2	3	4
1	Difference of means	—	0.234	0.3000	0.3975
	p-valor	—	<.001	<.001	<.001
2	Difference of means		—	0.0661	0.1636
	p-valor		—	.002	<.001
3	Difference of means			—	0.0975
	p-valor			—	<.001
4	Difference of means				—

	p-valor				—

Test Post-Hoc Games-Howell – AN

		1	2	3	4
1	Difference of means	—	-0.563	-0.905	-1.441
	p-valor	—	<.001	<.001	<.001
2	Difference of means		—	-0.341	-0.877
	p-valor		—	<.001	<.001
3	Difference of means			—	-0.536
	p-valor			—	<.001
4	Difference of means				—
	p-valor				—

Test Post-Hoc Games-Howell – AR

		1	2	3	4
1	Difference of means	—	-0.529	-0.856	-1.330
	p-valor	—	<.001	<.001	<.001
2	Difference of means		—	-0.327	-0.800
	p-valor		—	<.001	<.001
3	Difference of means			—	-0.474
	p-valor			—	<.001
4	Difference of means				—
	p-valor				—

Test Post-Hoc Games-Howell – AT

		1	2	3	4
1	Difference of means	—	-38.5	21.2	44.3
	p-valor	—	<.001	<.001	<.001
2	Difference of means		—	59.7	82.8
	p-valor		—	<.001	<.001
3	Difference of means			—	23.2
	p-valor			—	<.001
4	Difference of means				—
	p-valor				—

Test Post-Hoc Games-Howell – AV

		1	2	3	4
1	Difference of means	—	0.333	0.142	-0.525
	p-valor	—	.871	.988	.677
2	Difference of means		—	-0.191	-0.858
	p-valor		—	<.001	<.001
3	Difference of means			—	-0.666
	p-valor			—	<.001
4	Difference of means				—
	p-valor				—

Test Post-Hoc Games-Howell – AX

		1	2	3	4
1	Difference of means	—	814	1105	1398
	p-valor	—	<.001	<.001	<.001
2	Difference of means		—	291	584
	p-valor		—	<.001	<.001
3	Difference of means			—	293
	p-valor			—	<.001
4	Difference of means				—
	p-valor				—

Test Post-Hoc Games-Howell – BB

		1	2	3	4
1	Difference of means	—	-1.31	-1.681	-1.862
	p-valor	—	.023	.002	<.001
2	Difference of means		—	-0.373	-0.554
	p-valor		—	.024	<.001
3	Difference of means			—	-0.181
	p-valor			—	.553
4	Difference of means				—
	p-valor				—

Test Post-Hoc Games-Howell – BC

		1	2	3	4
1	Difference of means	—	8.12	22.9	25.21
	p-valor	—	<.001	<.001	<.001
2	Difference of means		—	14.8	17.09

		1	2	3	4
	p-valor		—	<.001	<.001
3	Difference of means			—	2.32
	p-valor			—	.663
4	Difference of means				—
	p-valor				—

Test Post-Hoc Games-Howell – BG

		1	2	3	4
1	Difference of means	—	0.385	0.3744	0.3554
	p-valor	—	.833	.845	.864
2	Difference of means		—	-0.0106	-0.0297
	p-valor		—	.686	.037
3	Difference of means			—	-0.0190
	p-valor			—	.437
4	Difference of means				—
	p-valor				—

Test Post-Hoc Games-Howell – CA

		1	2	3	4
1	Difference of means	—	121	948	1490
	p-valor	—	.005	<.001	<.001
2	Difference of means		—	827	1368
	p-valor		—	<.001	<.001
3	Difference of means			—	542
	p-valor			—	<.001
4	Difference of means				—
	p-valor				—

Test Post-Hoc Games-Howell – CD

		1	2	3	4
1	Difference of means	—	332	1196	1802
	p-valor	—	<.001	<.001	<.001
2	Difference of means		—	865	1471
	p-valor		—	<.001	<.001
3	Difference of means			—	606
	p-valor			—	<.001
4	Difference of means				—

	p-valor				—

Test Post-Hoc Games-Howell – CG

		1	2	3	4
1	Difference of means	—	0.758	0.928	1.535
	p-valor	—	<.001	<.001	.002
2	Difference of means		—	0.170	0.777
	p-valor		—	<.001	<.001
3	Difference of means			—	0.606
	p-valor			—	<.001
4	Difference of means				—
	p-valor				—

Test Post-Hoc Games-Howell – CK

		1	2	3	4
1	Difference of means	—	0.430	0.42308	0.3943
	p-valor	—	.767	.776	.811
2	Difference of means		—	-0.00702	-0.0358
	p-valor		—	.011	<.001
3	Difference of means			—	-0.0288
	p-valor			—	<.001
4	Difference of means				—
	p-valor				—

Test Post-Hoc Games-Howell – CN

		1	2	3	4
1	Difference of means	—	-256	2.54	7.99
	p-valor	—	.004	.848	.174
2	Difference of means		—	258.13	263.58
	p-valor		—	.003	.003
3	Difference of means			—	5.45
	p-valor			—	.543
4	Difference of means				—
	p-valor				—

Test Post-Hoc Games-Howell – CO

		1	2	3	4
1	Difference of means	—	160099	124968	186496
	p-valor	—	<.001	<.001	<.001
2	Difference of means		—	-35131	26397
	p-valor		—	<.001	.032
3	Difference of means			—	61528
	p-valor			—	.004
4	Difference of means				—
	p-valor				—

Test Post-Hoc Games-Howell – CP

		1	2	3	4
1	Difference of means	—	0.329	0.0405	-2.07
	p-valor	—	.887	1.000	.036
2	Difference of means		—	-0.2882	-2.40
	p-valor		—	<.001	<.001
3	Difference of means			—	-2.11
	p-valor			—	.004
4	Difference of means				—
	p-valor				—

Test Post-Hoc Games-Howell – CQ

		1	2	3	4
1	Difference of means	—	0.449	0.45194	0.44753
	p-valor	—	.757	.753	.758
2	Difference of means		—	0.00321	-0.00120
	p-valor		—	<.001	.537
3	Difference of means			—	-0.00442
	p-valor			—	<.001
4	Difference of means				—
	p-valor				—

Test Post-Hoc Games-Howell – CR

		1	2	3	4
1	Difference of means	—	0.465	0.4248	-0.210
	p-valor	—	.738	.789	.975
2	Difference of means		—	-0.0406	-0.676

	p-valor	—	.126	.008
3	Difference of means		—	-0.635
	p-valor		—	.015
4	Difference of means			—
	p-valor			—

Sample 2 at 25 keV

ANOVA one Factor (Fisher)

	F	gl1	gl2	p
E	75.9	3	90	<.001
G	102.2	3	90	<.001
H	24.9	3	90	<.001
I	37.8	3	90	<.001
M	137.9	3	90	<.001
P	26.7	3	90	<.001
Q	136.5	3	90	<.001
R	106.9	3	90	<.001
S	24.5	3	90	<.001
W	13.5	3	90	<.001
X	20.6	3	90	<.001
AA	200.9	3	90	<.001
AC	89.7	3	90	<.001
AD	77.4	3	90	<.001
AF	73.5	3	90	<.001
AI	181.6	3	90	<.001
AN	89.3	3	90	<.001
AR	95.2	3	90	<.001
AT	110.8	3	90	<.001
AV	49.5	3	90	<.001
AX	29.5	3	90	<.001
BB	43.3	3	90	<.001
BC	44.0	3	90	<.001
CA	38.3	3	90	<.001
CD	56.9	3	90	<.001
CG	73.6	3	90	<.001
CK	75.7	3	90	<.001
CN	56.6	3	90	<.001

CO	17.5	3	90	<.001
CP	62.2	3	90	<.001
CQ	45.5	3	90	<.001
CR	36.0	3	90	<.001

Test post hoc

Post-Hoc Games-Howell – E

		1	2	3	4
1	Difference of means	—	-29.1	-34.16	-87.3
	p-valor	—	<.001	<.001	<.001
2	Difference of means		—	-5.03	-58.1
	p-valor		—	<.001	.001
3	Difference of means			—	-53.1
	p-valor			—	.003
4	Difference of means				—
	p-valor				—

Post-Hoc Games-Howell – G

		1	2	3	4
1	Difference of means	—	-0.307	0.380	-0.975
	p-valor	—	<.001	<.001	<.001
2	Difference of means		—	0.686	-0.668
	p-valor		—	<.001	.002
3	Difference of means			—	-1.354
	p-valor			—	<.001
4	Difference of means				—
	p-valor				—

Post-Hoc Games-Howell – H

		1	2	3	4
1	Difference of means	—	-10.1	-1.54	-17.66
	p-valor	—	<.001	<.001	.012
2	Difference of means		—	8.52	-7.60

	p-valor		—	<.001	.421
3	Difference of means			—	-16.12
	p-valor			—	.022
4	Difference of means				—
	p-valor				—

Post-Hoc Games-Howell – P

		1	2	3	4
1	Difference of means	—	-4.17	-0.638	-9.10
	p-valor	—	<.001	<.001	.008
2	Difference of means		—	3.535	-4.93
	p-valor		—	<.001	.201
3	Difference of means			—	-8.46
	p-valor			—	.014
4	Difference of means				—
	p-valor				—

Post-Hoc Games-Howell – Q

		1	2	3	4
1	Difference of means	—	-22.5	-32.7	-59.3
	p-valor	—	<.001	<.001	<.001
2	Difference of means		—	-10.3	-36.8
	p-valor		—	<.001	<.001
3	Difference of means			—	-26.6
	p-valor			—	<.001
4	Difference of means				—
	p-valor				—

Post-Hoc Games-Howell – R

		1	2	3	4
1	Difference of means	—	0.401	0.138	-2.63
	p-valor	—	<.001	.023	<.001
2	Difference of means		—	-0.262	-3.03
	p-valor		—	.058	<.001

3 Difference of means			—	-2.77
p-valor			—	<.001
4 Difference of means				—
p-valor				—

Post-Hoc Games-Howell – S

	1	2	3	4
1 Difference of means	—	-7.48e+6	-9.92e+6	-5.78e+6
p-valor	—	<.001	<.001	.084
2 Difference of means		—	-2.44e+6	1.71e+6
p-valor		—	.106	.886
3 Difference of means			—	4.14e+6
p-valor			—	.292
4 Difference of means				—
p-valor				—

Post-Hoc Games-Howell – W

	1	2	3	4
1 Difference of means	—	-0.952	0.344	-1647
p-valor	—	<.001	<.001	.087
2 Difference of means		—	1.296	-1646
p-valor		—	<.001	.087
3 Difference of means			—	-1648
p-valor			—	.087
4 Difference of means				—
p-valor				—

Post-Hoc Games-Howell – X

	1	2	3	4
1 Difference of means	—	-0.0845	-0.229	-102
p-valor	—	.753	<.001	.029
2 Difference of means		—	-0.145	-102
p-valor		—	.355	.029
3 Difference of means			—	-102

	p-valor			—	.029
4	Difference of means				—
	p-valor				—

Post-Hoc Games-Howell – AA

		1	2	3	4
1	Difference of means	—	-0.442	0.0487	-0.542
	p-valor	—	<.001	.396	<.001
2	Difference of means		—	0.4902	-0.100
	p-valor		—	<.001	.172
3	Difference of means			—	-0.590
	p-valor			—	<.001
4	Difference of means				—
	p-valor				—

Post-Hoc Games-Howell – AC

		1	2	3	4
1	Difference of means	—	0.204	0.380	-0.273
	p-valor	—	<.001	<.001	.002
2	Difference of means		—	0.177	-0.476
	p-valor		—	<.001	<.001
3	Difference of means			—	-0.653
	p-valor			—	<.001
4	Difference of means				—
	p-valor				—

Post-Hoc Games-Howell – AD

		1	2	3	4
1	Difference of means	—	0.0566	0.0985	-0.168
	p-valor	—	<.001	<.001	.001
2	Difference of means		—	0.0419	-0.225
	p-valor		—	<.001	<.001
3	Difference of means			—	-0.267
	p-valor			—	<.001

4	Difference of means	—
	p-valor	—

Post-Hoc Games-Howell – AF

		1	2	3	4
1	Difference of means	—	-0.0415	-0.0671	0.0457
	p-valor	—	<.001	<.001	.021
2	Difference of means		—	-0.0257	0.0871
	p-valor		—	<.001	<.001
3	Difference of means			—	0.1128
	p-valor			—	<.001
4	Difference of means				—
	p-valor				—

Post-Hoc Games-Howell – AI

		1	2	3	4
1	Difference of means	—	0.380	0.0194	0.3429
	p-valor	—	<.001	.691	<.001
2	Difference of means		—	-0.3607	-0.0372
	p-valor		—	<.001	.688
3	Difference of means			—	0.3235
	p-valor			—	<.001
4	Difference of means				—
	p-valor				—

Post-Hoc Games-Howell – AN

		1	2	3	4
1	Difference of means	—	-0.207	0.736	-1.31
	p-valor	—	.002	<.001	<.001
2	Difference of means		—	0.943	-1.10
	p-valor		—	<.001	<.001
3	Difference of means			—	-2.04
	p-valor			—	<.001
4	Difference of means				—

	p-valor	—

Post-Hoc Games-Howell – AR

		1	2	3	4
1	Difference of means	—	-0.290	0.602	-1.158
	p-valor	—	<.001	<.001	<.001
2	Difference of means		—	0.892	-0.868
	p-valor		—	<.001	.001
3	Difference of means			—	-1.760
	p-valor			—	<.001
4	Difference of means				—
	p-valor				—

Post-Hoc Games-Howell – AT

		1	2	3	4
1	Difference of means	—	116	173.4	216.4
	p-valor	—	<.001	<.001	<.001
2	Difference of means		—	57.5	100.5
	p-valor		—	<.001	<.001
3	Difference of means			—	43.0
	p-valor			—	.006
4	Difference of means				—
	p-valor				—

Post-Hoc Games-Howell – AV

		1	2	3	4
1	Difference of means	—	-0.100	-0.0536	-4.66
	p-valor	—	<.001	.005	.001
2	Difference of means		—	0.0468	-4.56
	p-valor		—	.058	.001
3	Difference of means			—	-4.61
	p-valor			—	.001
4	Difference of means				—
	p-valor				—

Post-Hoc Games-Howell – AX

		1	2	3	4
1	Difference of means	—	-95.0	-397	14.6
	p-valor	—	<.001	<.001	.599
2	Difference of means		—	-302	109.6
	p-valor		—	.003	<.001
3	Difference of means			—	411.2
	p-valor			—	<.001
4	Difference of means				—
	p-valor				—

Post-Hoc Games-Howell – BB

		1	2	3	4
1	Difference of means	—	-0.988	-0.402	-0.739
	p-valor	—	<.001	<.001	.010
2	Difference of means		—	0.585	0.248
	p-valor		—	<.001	.597
3	Difference of means			—	-0.337
	p-valor			—	.402
4	Difference of means				—
	p-valor				—

Post-Hoc Games-Howell – BC

		1	2	3	4
1	Difference of means	—	57.9	66.47	22.3
	p-valor	—	<.001	<.001	.300
2	Difference of means		—	8.60	-35.6
	p-valor		—	.283	.045
3	Difference of means			—	-44.2
	p-valor			—	.012
4	Difference of means				—
	p-valor				—

Post-Hoc Games-Howell – CA

		1	2	3	4
1	Difference of means	—	-357	-498	747
	p-valor	—	.004	<.001	<.001
2	Difference of means		—	-142	1103
	p-valor		—	.604	<.001
3	Difference of means			—	1245
	p-valor			—	<.001
4	Difference of means				—
	p-valor				—

Post-Hoc Games-Howell – CD

		1	2	3	4
1	Difference of means	—	344	-351	1207
	p-valor	—	.002	<.001	<.001
2	Difference of means		—	-695	863
	p-valor		—	<.001	<.001
3	Difference of means			—	1558
	p-valor			—	<.001
4	Difference of means				—
	p-valor				—

Post-Hoc Games-Howell – CG

		1	2	3	4
1	Difference of means	—	-0.718	-1.212	0.678
	p-valor	—	<.001	<.001	.033
2	Difference of means		—	-0.494	1.396
	p-valor		—	<.001	<.001
3	Difference of means			—	1.889
	p-valor			—	<.001
4	Difference of means				—
	p-valor				—

Post-Hoc Games-Howell – CO

		1	2	3	4

		1	2	3	4
1	Difference of means	—	0.0476	0.0653	-0.0645
	p-valor	—	<.001	<.001	.008
2	Difference of means		—	0.0177	-0.1121
	p-valor		—	.004	<.001
3	Difference of means			—	-0.1298
	p-valor			—	<.001
4	Difference of means				—
	p-valor				—

Post-Hoc Games-Howell – CN

		1	2	3	4
1	Difference of means	—	66.4	71.98	81.21
	p-valor	—	<.001	<.001	<.001
2	Difference of means		—	5.58	14.81
	p-valor		—	.029	<.001
3	Difference of means			—	9.23
	p-valor			—	<.001
4	Difference of means				—
	p-valor				—

Post-Hoc Games-Howell – CK

		1	2	3	4
1	Difference of means	—	-0.00436	-0.0158	-0.0320
	p-valor	—	<.001	.002	.014
2	Difference of means		—	-0.0114	-0.0276
	p-valor		—	.026	.035
3	Difference of means			—	-0.0162
	p-valor			—	.352
4	Difference of means				—
	p-valor				—

Post-Hoc Games-Howell – CP

		1	2	3	4

		1	2	3	4
1	Difference of means	—	-0.299	0.0783	-14.8
	p-valor	—	<.001	.445	<.001
2	Difference of means		—	0.3776	-14.5
	p-valor		—	<.001	<.001
3	Difference of means			—	-14.9
	p-valor			—	<.001
4	Difference of means				—
	p-valor				—

Post-Hoc Games-Howell – CQ

		1	2	3	4
1	Difference of means	—	0.0193	0.02522	0.01583
	p-valor	—	<.001	<.001	<.001
2	Difference of means		—	0.00589	-0.00349
	p-valor		—	<.001	.512
3	Difference of means			—	-0.00939
	p-valor			—	.008
4	Difference of means				—
	p-valor				—

Post-Hoc Games-Howell – CR

		1	2	3	4
1	Difference of means	—	-0.0304	-0.03440	-4.32
	p-valor	—	<.001	<.001	.004
2	Difference of means		—	-0.00402	-4.29
	p-valor		—	.949	.004
3	Difference of means			—	-4.29
	p-valor			—	.004
4	Difference of means				—
	p-valor				—

Post-Hoc Games-Howell – M

		1	2	3	4
1	Difference of means	—	-23.4	-32.94	-39.81

		1	2	3	4
	p-valor	—	<.001	<.001	<.001
2	Difference of means		—	-9.55	-16.42
	p-valor		—	<.001	<.001
3	Difference of means			—	-6.87
	p-valor			—	.006
4	Difference of means				—
	p-valor				—

Post-Hoc Games-Howell – I

		1	2	3	4
1	Difference of means	—	0.557	-0.0678	-9.54
	p-valor	—	<.001	.835	.004
2	Difference of means		—	-0.6249	-10.10
	p-valor		—	<.001	.003
3	Difference of means			—	-9.48
	p-valor			—	.004
4	Difference of means				—
	p-valor				—
