# Supplementary material for: Radiomic Characterization of Breast Tissue from Breast CT Images Obtained with Synchrotron Beams
Source: Tomography. 2026 Aug 29;12(9):124. doi: 10.3390/tomography12090124 (PMC13611729; doi:10.3390/tomography12090124)
Supplement: Supplementary file 1 [file tomography-12-00124-s001.zip › S3 Leave-one-specimen-out cross-validation analysis supplementary.pdf]

Cross validation analysis in LDA

To further assess the potential influence of specimen-level dependence, we performed a leave-one-specimen-out cross-validation analysis using specimen-level separation, in which the classification model was evaluated while keeping specimens separated between training and validation subsets. The resulting classification performance was highly consistent with that obtained in the original analysis. The mean values of the radiomic features for the four tissue sub-types showed a high degree of consistency across the different specimen-based validation runs.

The high consistency of the group means across specimen-level validation runs provides additional evidence that the observed tissue-related patterns are not dominated by specimen-specific effects.

Discriminant analysis, samples 1, 2 and 3

Group means

	Perc 90	Kurt	IDM	LDE	Coars	Skew
1	43.705	3.036	0.030	8.511	0.565	0.984
2	54.677	2.862	0.100	8.094	0.182	0.963
3	81.074	2.899	0.110	7.740	0.123	0.947
4	84.509	2.890	0.560	7.393	0.107	0.934

Validation with sample 4

Group means

	Perc 90	Kurt	IDM	Skw	LDE	Coars
1	44.315	3.035	0.026	0.959	8.054	0.247
2	59.441	2.649	0.197	0.958	8.019	0.111
3	62.499	4.401	0.082	0.969	8.236	0.230
4	88.254	11.273	2.355	0.910	7.072	0.06

Discriminant analysis, samples 1, 2 and 4

Group means

	Perc 90	Kurt	IDM	Skw	LDE	Coars
1	43.601	3.038	0.033	0.984	9.298	0.553
2	54.599	2.969	0.134	0.964	8.116	0.194

3	80.498	3.015	0.045	0.946	7.710	0.133
4	85.540	2.932	0.568	0.934	7.421	0.120

Validation with sample 3

Group means

	Perc 90	Kurt	IDM	Skw	LDE	Coars
1	44.040	3.030	0.023	0.983	8.489	0.588
2	54.979	2.612	0.023	0.960	8.044	0.166
3	81.297	2.760	0.535	0.934	7.305	0.064
4	82.434	2.626	0.262	0.950	7.812	0.100

Discriminant analysis, samples 1, 3 and 4

Group means

	Perc 90	Kurt	IDM	Skw	LDE	Coars
1	44.169	3.032	0.024	0.971	8.284	0.428
2	57.048	2.631	0.08	0.959	8.031	0.135
3	69.877	3.744	0.149	0.962	8.079	0.182
4	85.144	8.010	1.655	0.920	7.175	0.073

Validation with sample 2

Group means

	Perc 90	Kurt	IDM	Skw	LDE	Coars
1	43.226	3.035	0.036	0.984	8.502	0.640
2	52.811	3.173	0.04	0.965	8.130	0.230
3	79.959	2.464	0.276	0.952	7.849	0.120
4	79.717	2.660	0.546	0.950	7.795	0.124

Discriminant analysis, samples 2, 3 and 4

Group means

	Perc 90	Kurt	IDM	Skw	LDE	Coars
1	43.855	3.033	0.028	0.976	8.362	0.505
2	55.463	2.834	0.05	0.961	8.068	0.171
3	72.471	3.415	0.182	0.960	8.020	0.166
4	83.245	5.884	1.216	0.932	7.416	0.093

Validation Sample 1

Group means

	Perc 90	Kurt	IDM	Skw	LDE	Coars
1	43.789	3.041	0.030	0.986	10.114	0.474
2	56.387	2.766	0.271	0.962	8.103	0.158
3	80.852	3.377	.106	0.942	7.619	0.142
4	93.516	3.306	0.599	0.911	6.910	0.116

General LDA paper

Group means

	Perc 90	Kurt	IDM	Skw	LDE	Coars
Fat	43.837	3.036	0.029	0.979	8.835	0.497
Glandular	55.715	2.815	0.036	0.962	8.078	0.167
Fiber	74.830	3.404	0.101	0.955	7.907	0.159
Microcalcificacions	85.584	5.297	1.076	0.927	7.301	0.098
